# Extracellular vesicles in atherosclerosis and vascular calcification: the versatile non-coding RNAs from endothelial cells and vascular smooth muscle cells

**DOI:** 10.3389/fmed.2023.1193660

**Published:** 2023-07-04

**Authors:** Fengyi Yu, Yingjie Duan, Chongmei Liu, Hong Huang, Xiangcheng Xiao, Zhangxiu He

**Affiliations:** ^1^Department of Nephrology, The First Affiliated Hospital, Hengyang Medical School, University of South China, Hengyang, Hunan, China; ^2^Department of Pathology, Yueyang People's Hospital, Yueyang, Hunan, China; ^3^Hengyang Medical School, The First Affiliated Hospital, Institute of Clinical Medicine, University of South China, Hengyang, Hunan, China; ^4^Department of Nephrology, Xiangya Hospital, Central South University, Changsha, Hunan, China

**Keywords:** atherosclerosis (AS), vascular calcification (VC), extracellular vesicles (EVs), non-coding RNAs (ncRNAs), uremia

## Abstract

Atherosclerosis (AS) is characterized by the accumulation of lipids, fibrous elements, and calcification in the innermost layers of arteries. Vascular calcification (VC), the deposition of calcium and phosphate within the arterial wall, is an important characteristic of AS natural history. However, medial arterial calcification (MAC) differs from intimal calcification and cannot simply be explained as the consequence of AS. Endothelial cells (ECs) and vascular smooth muscle cells (VSMCs) are directly involved in AS and VC processes. Understanding the communication between ECs and VSMCs is critical in revealing mechanisms underlying AS and VC. Extracellular vesicles (EVs) are found as intercellular messengers in kinds of physiological processes and pathological progression. Non-coding RNAs (ncRNAs) encapsulated in EVs are involved in AS and VC, including microRNAs (miRNAs), long non-coding RNAs (lncRNAs), and circular RNAs (circRNAs). The effects of ncRNAs have not been comprehensively understood, especially encapsulated in EVs. Some ncRNAs have demonstrated significant roles in AS and VC, but it remains unclear the functions of the majority ncRNAs detected in EVs. In this review, we summarize ncRNAs encapsulated in EC-EVs and VSMC-EVs, and the signaling pathways that are involved in AS and VC.

## 1. Introduction

Atherosclerosis (AS) is associated with systemic risk factors, including hypertension, hyperlipidemia, and diabetes mellitus, and is characterized by the accumulation of lipids, fibrous elements, and calcification in the innermost layers of arteries (1, 2). Several cell types are directly involved in the pathological progression of AS, such as endothelial cells (ECs), vascular smooth muscle cells (VSMCs), platelets, and foam cells (3). As a cellular monolayer lining the blood vessel wall, ECs first contact endogenous metabolite-related signals in the bloodstream, in which ECs function as danger signal sensors (4). ECs play an important role in inhibiting AS by regulating vascular tension and regulating inflammation (5). However, ECs can respond to oxidized low-density lipoprotein (ox-LDL) through different mechanisms, including EC dysfunction (6). Endothelial dysfunction is essential in the pathogenesis of AS (7–10), which releases inflammatory cytokines to VSMCs contributing to AS, including interleukin-1β (IL-1β), tumor necrosis factor-a (TNF-α), and transforming growth factor-β (TGF-β) (11, 12). VSMCs are the sole component of the center layer of the vessel wall, the tunica media (13). VSMCs are hypothesized whose principal function is contraction, so the contribution of VSMCs to AS has been greatly underestimated (14, 15). VSMCs have remarkable plasticity and reprogramming capacity during the complex AS process, including contractile phenotype, macrophage-like, foam cell-like, osteochondrogenic-like, myofibroblast-like, and mesenchymal stem cell-like (11, 16). The hypothesis that VSMCs possess a “transitional stage between smooth muscle and foam cells” has been demonstrated, which highlights the importance of VSMC phenotypic modulation in AS (17–19). The switching of VSMCs to macrophage-like cells may be driven by lipids accumulation which was Krüppel-like factor 4 (KLF4) dependent (20, 21).

**Figure 1:**
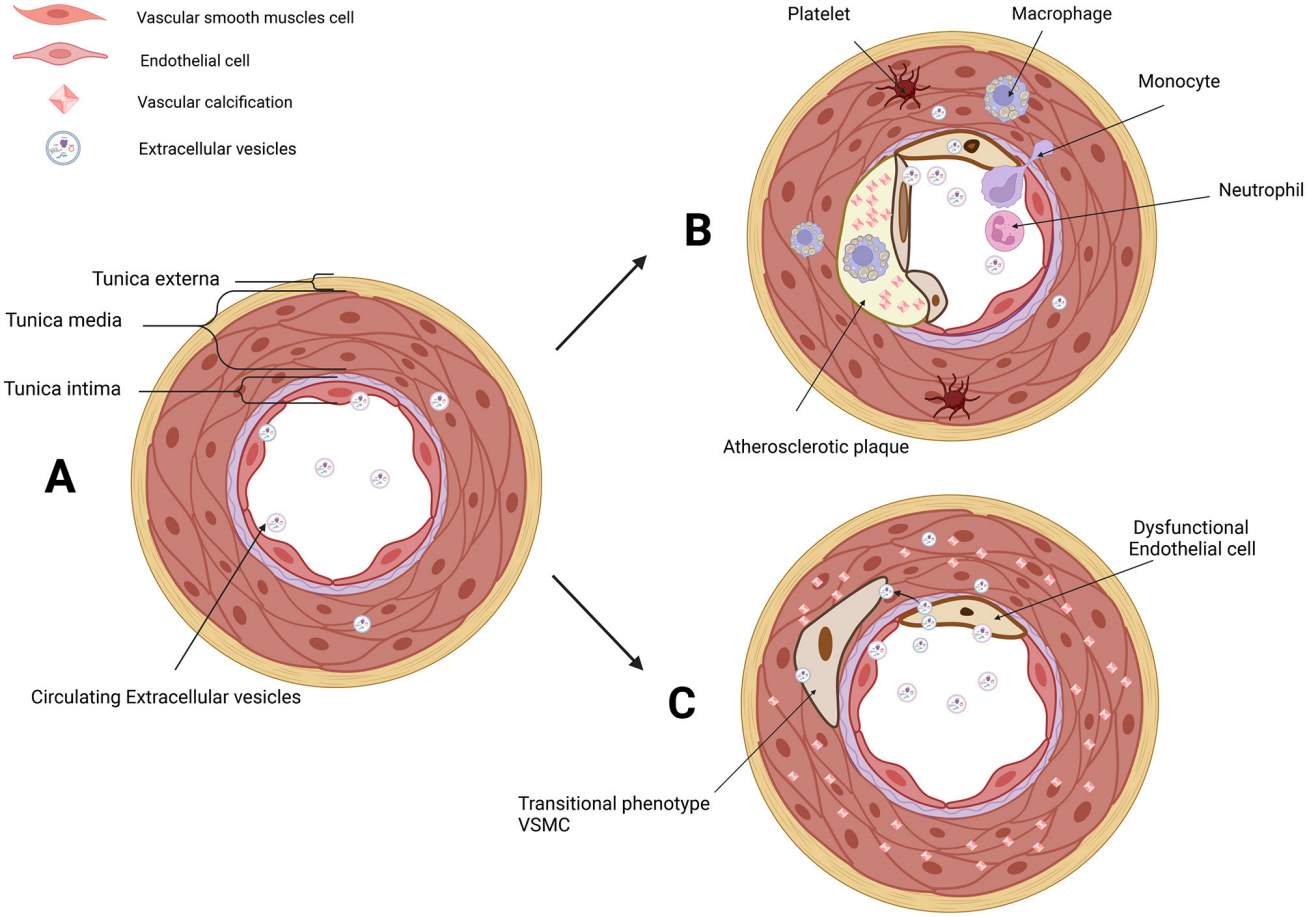
EVs in the microenvironment of IAC in AS and MAC. **(A)** Three layers of arteries: tunica externa, tunica media (mainly composed of VSMCs), and tunica intima (composed of ECs and subendothelial layer). VSMCs and ECs are both the donor cells and recipient cells in the microenvironment of vascular. Circulating EVs in serum and EVs derived from VSMCs and ECs have been involved in cellular communication. **(B)** Platelet, monocyte, macrophage, neutrophil, transitional VSMCs, and dysfunctional ECs are involved in AS and IAC. EVs derived from VSMCs and ECs are novel messengers in the microenvironment of AS. **(C)** Dysfunctional ECs and transitional VSMCs are involved in MAC. EVs derived from VSMCs and ECs are novel messengers in the microenvironment of MAC. EVs, extracellular vesicles; IAC, intimal arterial calcification; AS, atherosclerosis; MAC, medial arterial calcification; VSMCs, vascular smooth muscle cells; ECs, endothelial cells. Created with BioRender.com.

Vascular calcification (VC), the deposition of calcium and phosphate within the arterial wall, is an important part of AS natural history and an independent predictor of cardiovascular morbidity and mortality (22–25). VC occurs in both the intimal and medial layers of the arteries, and intimal arterial calcification (IAC) is mainly involved in the calcification of atherosclerotic lesions (26, 27). Calcifying vascular cells are derived from local VSMCs, especially in IAC (28). On the contrary, medial arterial calcification (MAC) is a chronic systemic vascular disorder distinct from AS, in which its hallmark is the dissemination and progressive precipitation of calcium phosphate within the medial layer (29). MAC differs in several ways from the IAC seen in atherosclerotic lesions, including cellular aging linked to mechanical stress cooperated and buffered by ECs (27, 30, 31). Initially, VC was regarded as a passive degenerative process, and now VC is elucidated to be a multifactorial process through the phenotypic modulation of VSMCs (26, 32–36). It means that ECs have a potential role in MAC. Correspondingly, endothelial dysfunction under aging or injured condition promoted VC (37–39). More importantly, it has been demonstrated that endothelial dysfunction aggravated MAC *in vivo* (40, 41). Moreover, extracellular vesicles (EVs) found a novel messenger in the cellular crosstalk between ECs and VSMCs in VC (42, 43) (Figure 1).

Extracellular vesicles, as the generic term for particles, are derived from cells and are delimited by a lipid bilayer (44). Non-coding RNAs (ncRNAs), messenger RNAs (mRNAs), and lipids encapsulated in EVs can involve in intracellular function regulation and intercellular communication. It has been reported that there were a growing number of studies about microRNAs (miRNAs) encapsulated in EVs related to VC (45–50). Nevertheless, there were few special studies aimed to summarize the miRNAs encapsulated in EVs during intercellular communication. Therefore, considering the prospect of cellular communication through EVs, first, we propose to summarize that the miRNAs have appeared in EVs derived from ECs (EC-EVs) and EVs derived from VSMCs (VSMC-EVs) in the AS and VC processes. Subsequently, we will discuss the potential prospect of long non-coding RNAs (lncRNAs) and circular RNAs (circRNAs) in EC-EVs and VSMC-EVs during AS and VC formation.

## 2. The flexibility and versatility of miRNAs encapsulated in EC-EVs in AS and VC

The emerging role of EVs and intercellular communication has gained momentum in the past few years. In the process of biological message transferring from ECs to other cells, the presence of EVs has been verified in numerous physiological and pathological pathways, including AS and VC.

### 2.1. miRNAs encapsulated in EC-EVs regulate the process of AS

It has been shown that miRNAs encapsulated in EC-EVs exerted atheroprotective or proatherogenic function in several studies. The differential expression of miRNAs encapsulated in EC-EVs during AS was analyzed by miRNA-Sequence (miRNA-seq) (51, 52), providing a reasonable prospect to illuminate the detailed mechanisms miRNAs are involved in. Here, miRNAs encapsulated in EC-EVs including miR-92a-3p, miR-155-5p, miR-505-3p, miR-19b-3p, and miR-4306 promoted AS, while miR-126-5p and miR-145-5p inhibited AS process (53–61).

Xiang et al. (51) performed miRNA-Seq analysis and found that EVs derived from lipopolysaccharide (LPS)-stimulated human umbilical vein endothelial cells (HUVECs) promoted human aortic smooth muscle cell (HASMC) proliferation and migration, in which four upregulated miRNAs (including miR-27a-3p, miR-126-5p, miR-155-5p, and miR-365b-3p) and nine downregulated miRNAs (including let-7b-5p, miR-10a-5p, miR-10b-5p, miR-21-3p, miR-30a-5p, miR-92a-3p, miR-125a-3p, miR-143-3p, and miR-181a-2-3p) participated in various aspects of atherosclerotic lesion formation. In another RNA-seq research, a significant upregulation of 49 miRNAs (including miR-19b-3p and miR-126-5p) and downregulation of 19 miRNAs in extra-virgin olive-induced HUVECs-EVs compared to sunflower oil-induced HUVECs-EVs were observed, which closely linked to the development of AS (52). The finding of higher expression of miR-126-5p was detected in both studies (51, 52). It is no coincidence that miR-126-5p was found to have a linear relationship with coronary calcium in outpatients with AS (53). However, the upregulation or transfection of miR-126-5p promoted ECs proliferation by the suppression of delta-like 1 homolog (Dlk1) and modulation of nuclear factor-κB (NF-κB)-mediated serine/threonine kinase (AKT) signaling pathway (62, 63). On the contrary, the upregulation or transfection of miR-126-5p limited ox-LDL-induced VSMC (ox-LDL-VSMC) proliferation and migration as an atheroprotective miRNA targeting high-mobility group box 1 (HMGB1) (64). In ECs and VSMCs, miR-126-5p showed opposite regulation: miR-126-5p promoted ECs proliferation and inhibited VSMCs proliferation and migration. From above, we suppose that miR-126-5p encapsulated in EC-EVs has a double atheroprotective regulation mechanism when recipient cells assimilate it: it promoted ECs proliferation and inhibited VSMCs proliferation and migration.

MiR-155, named miR-155-5p now according to miRNA online database miRBase (https://www.mirbase.org/), was upregulated according to the profile of miRNAs in EC-EVs (51) and was also enriched in ox-LDL-HUVEC-EVs (54). By enhancing monocyte activation toward proinflammatory M1 macrophages, miR-155 showed a proatherogenic function and was negatively regulated by Krüppel-like factor 2 (KLF2) in an experimental mouse model (54). MiR-155-5p has emerged as a significant component in AS (65). In senescent HUVECs, upregulated miR-155-5p mediated endothelial dysfunction through NF-κB/endothelial nitric oxide synthase (eNOS) axis and inhibited the proliferation of ECs by targeting Ras homolog enriched in brain (RHEB) expression (66–68). In VSMCs, miR-155-5p inhibited the viability of VSMCs and induced VSMCs phenotypic switching by inhibiting cyclic guanosine monophosphate (cGMP)-dependent protein kinase 1 (PKG1) (69, 70). In both ECs and VSMCs, miR-155-5p inhibited the proliferation and migration in AS by inhibiting AKT1 (71). MiR-155-5p encapsulated in EC-EVs, as distinct from miR-126-5p, was demonstrated as an element of inflammatory signal transduction in AS.

Additionally, miR-505, called miR-505-3p at present, is also highly expressed in exosomes from ox-LDL-HUVEC, which was finally proved to be a proatherogenic factor by inducing neutrophil extracellular trap (NET) formation *in vitro* (55). MiR-505-3p markedly inhibited the proliferation, migration, and tube formation capacity of HUVECs by targeting vascular endothelial growth factor A (VEGFA) (72, 73). Therefore, circulating miR-505-3p was a prognostic biomarker of endothelial dysfunction and inflammation in hypertension patients (74, 75). However, the biological function of miR-505-3p in VSMCs has not been reported, leaving to further investigation.

MiR-19b encapsulated in HUVECs-EVs mentioned above (52), the previous name of miR-19b-3p, reduced HUVECs migration and damaged lymphatic system function by negatively regulating TGF-βRII expression (56, 76). MiR-19b-3p in endothelial microparticles (MPs) exaggerated AS by targeting the suppressor of cytokine signaling 3 (SOCS3) (77). The upregulation of miR-19b-3p in ECs contributed to endothelial dysfunction by inhibiting syndecan-1 mRNA and protein expression (78–80). MiR-19b-3p exerted an inflammatory effect on the vascular microenvironment and promoted human retinal microvascular ECs apoptosis through SOCS6-mediated Janus kinase 2 (JAK2)/signal transducer and activator of transcription 3 (STAT3) axis (81, 82). Similarly, miR-19b-3p reduced hypoxia-induced HUVECs apoptosis, autophagy, and inflammation by the inhibition of hypoxia-induced factor-1 (HIF-1α) (83). MiR-19b-3p was increased by more than 3-fold in LPS-ECs (84). Inhibition of miR-19b-3p reduced TNF-α and IL-1β expression levels in ox-LDL-HUVEC and AS mice (85) and mitigated endothelial dysfunction (86). Overexpression of miR-19b-3p in human aortic endothelial cells (HAECs) caused intracellular reactive oxygen species accumulation, promoting apoptosis (87). However, Li et al. adopted a different perspective toward the role of miR-19b-3p in AS (88, 89). MiR-19b-3p inhibited HUVECs proliferation, migration, and angiogenesis targeting STAT3, which may, however, delay unstable plaque progression in patients with unstable angina (88, 89). This opinion was contrary to the mentioned studies (85–87). This was not the only case, Tang et al. (90) demonstrated that miR-19b-3p attenuated TNF-α-induced ECs apoptosis. We speculate that this biological difference might be due to the different stimuli (LPS, TNF-α, and so on), the different types of ECs (HAECs and HUVECs), and different periods of AS.

Furthermore, miR-4306 encapsulated in EVs derived from ox-LDL condition (ox-LDL-EVs) from human coronary artery vascular endothelial cells (HCAECs) upregulated the AKT/NF-κB signaling pathway enhancing the lipid formation of human monocyte-derived macrophages (HMDMs), while miR-4306 encapsulated in ox-LDL-EVs from HMDMs inhibited capillary tubule formation of HCAECs *in vitro* (57). Wang et al. (91) found that miR-4306 plays a key role in urban particulate matter-induced endothelial damage. As a result, miR-4306 manifested promotion in AS.

The previously reported MiR-92a-3p, named miR-92 or miR-92a before, performed a different function with its recipient cells during AS (51). MiR-92a-3p encapsulated in EVs from HUVEC treated with TNF-α or subjected to shear flows could be transported to macrophages to suppress KLF4, leading to the atheroprone phenotypes of macrophage in AS (58). The progression of AS was associated with a maladaptive form of angiogenesis which contributed to plaque disruption and intraplaque hemorrhage (92). Independent atherosclerotic stimuli (ox-LDL and IL-6) increased miR-92a-3p expression in donor HCAEC-EVs, promoting an angiogenic phenotype in the recipient ECs through the downregulation of thrombospondin 1 (THBS1) (59). Meaningfully, miR-92a-3p encapsulated in HCAEC-EVs increased in a balloon injury rat model and inhibited the atheroprotective expression of KLF2 (60). Uremic toxins accumulation upregulated the expression level of miR-92a-3p in ECs, contributing to endothelial dysfunction (93). MiR-92a-3p overexpression inhibited VSMCs proliferation and migration, while miR-92a-3p inhibition promoted the proliferation and migration of VSMCs via the KLF4 pathway (94, 95). MiR-92a-3p encapsulation in EC-EVs demonstrated a double effect on AS when recipient cells assimilate it: it promoted ECs phenotype switching while it inhibited VSMCs proliferation and migration.

MiR-143 (miR-143-3p) and miR-145 (miR-145-5p) encapsulated in KLF2-transduced or shear-stress-stimulated HUVEC-EVs induced an atheroprotective HASMC phenotype and reduced AS in the aorta of Apolipoprotein E Knock-Out (ApoE^(−/−)^) mice (61), supporting the bioinformatic prediction of Xiang et al. (51). MiR-143-3p and miR-145-5p have been reported frequently, their roles in AS and VC are discussed later.

In brief, numerous studies show plenty of potential EC-EV-miRNAs that are involved in the progress of AS and illustrate the multiple signaling pathways. Particularly, miR-126-5p was upregulated in EC-EVs in atherogenic condition, while previous studies showed its atheroprotective effect, addressing the complicated role of miR-126-5p in the AS process. Further studies are essential to explore the distribution of miR-126-5p between EVs and cells, and how biological function diversities are feasible. In addition, miR-155-5p promoted AS and KLF2 downregulated the expression of miR-155-5p showing its atheroprotective role. Correspondingly, the suppression of KLF2 by miR-92a-3p and the suppression of KLF4 by miR-92a-3p lead to AS. Additionally, the upregulation of miR-143-3p and miR-145-5p encapsulated in EC-EVs was transduced by KLF2 and showed an atheroprotective effect. KLF2, a shear-responsive transcription factor, was critical because it played a major role in exerting atheroprotective effects in ECs, which promised a novel target for AS prevention and treatment (96, 97). Similarly, KLF4, as a therapeutic target of atherosclerosis, regulates ECs inflammation, VSMC phenotypic transformation, and foam cell formation during the process of AS (98). Several miRNAs encapsulated in EC-EVs that regulate the process of AS are summarized in Table 1 and Supplementary Table 1.

**Table 1:** miRNAs encapsulated in EC-EVs regulate the process of AS.

**miRNA**	**EVs origin**	**Stimulation**	**Expression**	**Function**	**References**
miR-126-5p	HUVEC	LPS	Up	Various aspects of AS	(51)
miR-155-5p	HUVEC	LPS	Up	Various aspects of AS	(51)
miR-143-3p	HUVEC	LPS	Down	Various aspects of AS	(51)
miR-92a-3p	HUVEC	LPS	Down	Various aspects of AS	(51)
miR-126-5p	HUVEC	Extra-virgin-olive	Up	Closely linked to AS	(52)
miR-19b-3p	HUVEC	Extra-virgin-olive	Up	Closely linked to AS	(52)
miR-155-5p	HUVEC	ox-LDL	Up	Promoted AS	(54)
miR-505-3p	HUVEC	ox-LDL	Up	Promoted AS	(55)
miR-19b-3p	HUVEC	miR-19b-3p mimic	Up	Promoted AS	(56)
miR-4306	HCAEC	ox-LDL	Up	Promoted AS	(57)
miR-92a-3p	HUVEC	TNF-α/shear flows	Up	Promoted AS	(58)
miR-92a-3p	HCAEC	ox-LDL and IL-6	Up	Promoted angiogenesis	(59)
miR-92a-3p	HCAEC	Chrysin	Down	Promoted AS	(60)
miR-143-3p	HUVEC	KLF2 /shear-stress	Up	Inhibited AS	(61)
miR-145-5p	HUVEC	KLF2 /shear-stress	Up	Inhibited AS	(61)

EVs, extracellular vesicles; HUVEC, human umbilical vein endothelial cell; HCAEC, human coronary artery vascular endothelial cell; LPS, lipopolysaccharide; AS, atherosclerosis; ox-LDL, oxidized low-density lipoprotein; TNF-α, tumor necrosis factor-a; IL-6, interleukin-6; KLF2, Krüppel-like factor 2.

### 2.2. miRNAs encapsulated in EC-EVs regulate the process of VC

We have demonstrated for the first time that high phosphate (HP)-induced EVs from HUVECs directly promoted and accelerated VC through astrocyte elevated gene-1 (AEG-1) (99). Furthermore, miRNAs encapsulated in EC-EVs demonstrated significant messengers in the biogenesis of VC. Lin et al. (100) found that miR-670-3p encapsulated in EC-EVs stimulated by HP could target insulin-like growth factor-1 (IGF-1) resulting in potentiating the VC process *in vitro* and *in vivo*. Freise et al. applied EC-EVs with a miR-221 (miR-221-3p) inhibitor which provoked a distinct reduction of VSMC calcification, and miR-143 or miR-145 mimics also provoked a distinct reduction of VSMC calcification (101). MiR-29b (miR-29b-3p) and miR-126-5p in EC-EVs also negatively regulated VC (102, 103). Additionally, we detected the profile of miRNAs in HP-EVs from HUVECs by RNA-seq, finding that miR-3182 increased while 13 known miRNAs decreased in HP-EVs from HUVECs (104), indicating the potential function of miRNAs encapsulated in EC-EVs during VC.

MiR-29b-3p was named miR-29b previously according to miRBase. Wang *et al*. confirmed miR-29b-3p encapsulated in endothelial exosomes mediated anti-calcification and was associated with C-X-C motif chemokine receptor 6 (CXCR6) (102). Similarly, miR-29b-3p downregulated VSMC calcification by targeting matrix metalloproteinase 2 (MMP2) and Wnt family member 7B (Wnt7b)/β-catenin signal (105–107). The expression of miR-29b in the coronary artery calcification group was significantly lower than that in the control group, indicating that decreased miR-29b-3b level may be a coronary artery calcification risk factor (108). However, it is a paradox that Fang *et al*. found that miR-29b was increased in calcific aortic tissue and the inhibition of miR-29b mitigated aortic calcification in tissues of calcific aortic valve diseased rats via directly targeting TGF-β3 (109). Additionally, inorganic phosphorus (Pi) stimulation increased miR-29b-3p expression and overexpression of miR-29b-3p facilitated Pi-induced VSMC calcification (110). Intriguingly, the level of miR-29b shows no statistical significance in VSMCs during the exposure of 3 mM Pi, compared with normal VSMCs (111). Taken together, miR-29b-3p encapsulated in EC-EVs inhibited VSMC calcification, while the role of miR-29b-3p in VSMC calcification is still ambiguous. Considering specific distribution, further studies explaining the relationship between miR-29b-3p and VC are required in the future.

Freise et al. (101) revealed that three miRNAs (miR-221, miR-222, and miR-126) were significantly enhanced in uremic toxins-induced-EVs from human ECs. Subsequently, VSMC calcification was inhibited by a miR-221 inhibitor and a stronger reduction with a miR-222 inhibitor. Both miR-221 and miR-222 contribute to the atherogenic calcification of VSMCs (112). Correspondingly, increased calcium deposition was observed in the combined treatment of miR-221 and miR-222 that mimics synergistically but not in individual treatments, with significant changes in ectonucleotide phosphodiesterase 1 (Enpp1) and Pit-1 expression (113).

By contrast, Freise et al. (101) confirmed that the levels of miR-143 and miR-145 were significantly decreased during VSMC calcification. MiR-143 and miR-145 can both inhibit AS process as mentioned above (61). Similarly, an individual transfection with miR-143 or miR-145 mimics provoked a distinct reduction of VSMC calcification and a stronger reduction in a concomitant transfection (101). Massy et al. (114) suggested that decreasing levels of miR-143, miR-145, and miR-126, and increasing levels of miR-223 were potential biomarkers of vascular calcification associated with chronic kidney disease (CKD). Elevated Pi significantly downregulated miR-143 and miR-145 and upregulated miR-223 during VSMCs calcification *in vitro* and in ApoE^(−/−)^ mice (111, 115).

In conclusion, miR-143-3p and miR-145-5p inhibited VC, whereas miR-221-3p and miR-222-3p potentiated VC.

Based on Freise et al. (101) and Massy et al. (114) studies, the expression of miR-126 was found to be adverse, which probably was attributed to the different distribution of miR-126 among EC-EVs, VSMCs, and serum of CKD mice. Massy et al. (114) detected that the expression of miRNAs (including miR-126, miR-143, miR-145, and miR-223) in the endothelium or media of the thoracic and abdominal aorta is different. For example, miR-126 expression of VSMCs in the upper abdominal aorta is equal to ECs, lower than ECs in the low thoracic and low abdominal aorta, and higher than ECs in the upper thoracic (114). However, it must be remembered that miR-126 is known as miR-126-3p, and miR-126^*^ is referred to as miR-126-5p. Due to some reason, the specific sequence of miR-126 was not shown in all studies. As a result, miR-126 refers to miR-126-3p as default. It suggested that miR-126 can be considered a potential cardiovascular risk and VC biomarker in cardiovascular disease (CVD) and cerebral troubles associated with CKD (53, 114, 116, 117). Additionally, miR-126 expression is increased in CKD and ApoE^(−/−)^ mice aorta (118). Furthermore, miR-126 markedly enhanced the regeneration of vascular smooth muscle (119). In the progress of Pi-induced HASMCs calcification, miR-126 was involved in the Wnt/β-catenin signaling pathway (120). However, miR-126-5p encapsulated in HUVEC-EVs stimulated with advanced glycation end-products led to the reduction of calcium deposition by blocking the smad1/5/9 signaling pathway both *in vitro* and in a mouse model (103). In short, the biological effects are different between miR-126-3p and miR-126-5p. The upregulation of miR-126-3p encapsulated in EC-EVs probably enhances VC, while the upregulation of miR-126-5p encapsulated in EC-EVs prohibits VC. However, the versatility of miR-126-5p is still worth exploring because of its atherogenic function. Thus, it is necessary to validate these results in further study.

To determine whether the HP condition altered the profile of miRNAs, we (104) detected the upregulation expression of has-miR-3182, while the downregulation expression of 13 known hsa-miRs (including miR-10a-5p, miR-10b-5p, miR-30a-3p, miR-30a-5p, miR-30c-2-3p, miR-99b-5p, miR-143-3p, miR-193b-5p, miR-365a-5p, miR-486-5p, miR-941, and miR-7706) in the HP-EC-EVs group compared to the PBS-EC-EVs group by miRNA-seq bioinformatics analysis. Target mRNAs for hsa-miR-3182 included acyloxyacyl hydrolase (AOAH), nucleotide-binding oligomerization domain containing 2 (NOD2), and RNA-binding motif protein 47 (RBM47), implying that miR-3182 might be a novel miRNA in an HP-induced VC. In addition, accumulating evidence showed that AOAH, NOD2, and RBM47 were also related to AS, which has a close connection with VC (121–124). Interestingly, miR-10a-5p, miR-10b-5p, miR-30a-5p, and miR-143-3p were also downregulated and discovered in accordance with the mentioned research (50), indicating the protective roles of these miRNAs in the cardiovascular system. Correspondingly, miR-30a-5p, known as miR-30a, attenuated osteoblast maturation by suppressing runt-related transcription factor 2 (Runx2) and smad1/5 (125). Nevertheless, miR-30a was increased during osteogenic processes and the progression of VC (126–129). MiR-143-3p, which was referred to as miR-143, has been reported as a protective factor in AS and VC. MiR-486-5p, called miR-486 previously, increased in the calcification of aortic valve, promoting osteoblastic biomarkers Runx2, osterix (Osx), and calcium deposition of human aortic valve interstitial cells (AVICs) (130, 131). A further novel finding was that we discovered another 34 novel hsa-miRNAs, which included 31 downregulated miRNAs and 3 upregulated miRNAs. In the future, it is essential to confirm our data through *in vitro* and *in vivo* experiments to take a step forward.

We have discussed the ambiguous role of miR-29b-3p, anti-calcification roles of miR-143-3p, miR-145-5p, and miR-126-5p, and pro-calcification roles of miR-221-3p, miR-222-3p, and miR-126-3p. Overall, the results above show a novel perspective in intercellular communication during VC: miRNAs encapsulated in EC-EVs potentiate an anti-calcification or pro-calcification effect when transmitted to VSMCs. Several miRNAs encapsulated in EC-EVs that regulate the process of VC are summarized in Table 2 and Supplementary Table 2.

**Table 2:** miRNAs encapsulated in EC-EVs regulate the process of VC.

**miRNA**	**EVs origin**	**Stimulation**	**Expression**	**Function**	**References**
miR-670-3p	Mice EC	HP	Up	Promoted VC	(100)
miR-221-3p	Human EC	Urea and indoxyl sulfate	Up	Promoted VC	(101)
miR-222-3p	Human EC	Urea and indoxyl sulfate	Up	Promoted VC	(101)
miR-126-3p	Human EC	Urea and indoxyl sulfate	Up	No effect on VC	(101)
miR-143-3p	Human EC	Urea and indoxyl sulfate	Down	Inhibited VC	(101)
miR-145-5p	Human EC	Urea and indoxyl sulfate	Down	Inhibited VC	(101)
miR-29b-3p	Mouse AEC	Transverse aortic constriction	Down	Inhibited VC	(102)
miR-126-5p	HUVEC	Advanced glycation end-products	Up	Inhibited VC	(103)
miR-3182	HUVEC	HP	Up	Further researched required	(104)
miR-99b-5p	HUVEC	HP	Down	Further researched required	(104)

EVs, extracellular vesicles; EC, endothelial cell; AEC, aortic endothelial cell; HUVEC, human umbilical vein endothelial cell; HP, high phosphate; VC, vascular calcification.

## 3. The flexibility and versatility of miRNAs encapsulated in VSMC-EVs in AS and VC

The phenotype change from the contractible type to the synthetic type of VSMCs plays a crucial role in the biogenesis of AS, VC, and vascular dysfunction. VSMCs are one of the main cells implicated in AS, retaining remarkable plasticity (132). VSMCs can adopt alternative phenotypes resembling foam cells contributing to AS (133). VSMCs are also key players in VC, which transform into calcifying VSMCs and secrete mineralizing EVs that form microcalcifications, subsequently increasing plaque instability and consequential plaque rupture (134). In this review, we summarized miRNAs encapsulated in VSMC-EVs in the process of AS and VC.

### 3.1. miRNAs encapsulated in VSMC-EVs regulate the process of AS

The function and number of VSMCs in AS plaques according to lineage-tracing studies have been greatly underestimated (135). The majority of research on EVs that mediate cellular communication between VSMCs and other cell types has focused on foam cells, especially EVs from foam cells. As far as miRNAs are concerned, a couple of studies have illuminated the atherogenic function of miRNAs encapsulation in EVs from foam cells to VSMCs, including miR-19b-3p, miR-21-3p, miR-106a-3p, and miR-186-5p (136–139). Here, we aimed to review miRNAs encapsulated in VSMC-EVs in the progression of AS.

Gonzalo et al. (140) demonstrated first that MPs derived from human coronary artery smooth muscle cells (HCASMCs) contain miRNAs. Moreover, the packaging of both miR-143-3p and miR-222-3p was decreased, which is considered a response to hypercholesterolemia stress in HCASMC and conditioned medium of AS (140). Thus, it is conceivable that miR-143-3p and miR-222-3p in HCASMC-MPs would be a source of cardiovascular biomarkers. In 2009, Boettger *et al*. defined the miR-143/145 gene cluster as a major regulator of the contractile phenotype of VSMC (141). In 2012, Hergenreider et al. (61) elucidated a novel pathway linking endothelial KLF2 expression to VSMC phenotype regulating miR-143/145 and provided an intercellular communication mediated by miR-143/145 encapsulated in EC-EVs. In 2020, Chung et al. (142) demonstrated that miR-143 and miR-145 downregulated Ras and Ras homolog family member A (RhoA) expressions, which potentially prevented AS. Dégano et al. (143) identified that miR-143-3p was independently associated with time-to-coronary events and that a higher expression in healthy individuals was related to less risk of an incident coronary artery disease (CAD) event at 10 years. By contrast, González et al. (144) suggested that miR-143-3p may be implicated in carotid plaque instability by the modulation of IGF-IIR, contributing to AS.

The characteristics of miR-222-3p in the process of AS were subtle, which were different from the ones in the process of VC, as we summarized in EC-EVs. In 2010, Dentelli *et al*. demonstrated that miR-222 acted as an anti-angiogenic miRNA that negatively regulated STAT5A, providing a promising perspective to prevent the biogenesis of AS (145). Atherosclerotic plaque rupture exhibited a decrease in miR-221/222 (146). Both miR-221-3p and miR-222-3p decreased significantly in patients with AS, indicating that the downregulation of these two miRNAs may be connected with AS (147). In addition, miR-222 was involved in VSMCs proliferation and upregulated by Ang II in promoting ECs migration and inflammation (148). In 2015, Xue et al. (87) unveiled miR-221/222-induced EC dysfunction by the suppression of peroxisome proliferator-activated receptor-γ coactivator-1α (PGC-1α) in AS. Taraldsen et al. (149) observed that circulating levels of miR-92a-3p, miR-221-3p, and miR-222-3p were associated with baseline coronary necrotic core volume. We speculate that the duality of miR-221-3p and miR-222-3p is due to the different distribution of VC and that miR-221-3p and miR-222-3p probably play key roles in MAC.

Efforts would be essential to understand the conflicting role of miR-222-3p in AS. As a result, increasing number of evidence is required to address its time-based and spatial location in ECs, VSMCs, and EVs. The protective function of miR-143-3p seemed more specific than miR-222-3p in AS. Nevertheless, further studies are required to elucidate the relationship between the functional and distributional diversity of miR-143-3p and miR-222-3p encapsulated in EV.

Zheng et al. (150) found miR-155 encapsulated in KLF5-induced exosomes from VSMCs to ECs, which destroyed the integrity of endothelial barriers, resulting in AS. This result is consistent with the research mentioned above that elevated miR-155 enhanced AS progress (54, 151–154). González et al. (144) suggested that miR-155-5p might be implicated in plaque instability by targeting AKT, contributing to AS. MiR-155 activated the NLRP3 by regulating NF-κB in the AS progression of ApoE^(−/−)^ mice (155). However, evidence showed that miR-155 exerted inhibition on AS in ApoE^(−/−)^ mice (156). Park et al. (157) suggested that miR-155 was a novel negative regulator in the soluble guanylyl cyclase (sGC)/cGMP pathway, providing a novel therapeutic target for AS. MiR-155 attenuated ox-LDL-induced apoptosis in various cells by targeting p85α, revealing its therapeutic effect in AS (158). miR-155 was implicated in several signaling pathways in AS mouse models, as shown by the evidence. In the future, the distinction and differential expression of miR-155 in different conditions still need to be elucidated, and potential signaling pathways associated with AS also need to be uncovered.

Gonzalo et al. (159) discovered that atherogenic lipoproteins significantly reduced miR-15b-5p, −24-3p, −29b-3p, −130a-3p, −143-3p, −146a-3p, −222-3p, and −663a in microvesicles (MVs) released by HCASMC. MiR-143-3p, miR-222, and miR-29b have been discussed above. MiR-24-3p and miR-130a-3p were also reduced in circulating MVs from familial hypercholesterolemia (FH) patients. In addition, the expression of miR-130a-3p was reduced in uremia toxin indoxyl sulfate-induced HCAEC-EVs (160). Downregulation of miR-130a-3p expression leads to endothelial barrier dysfunction in the development of AS (161). Paradoxically, serum miR-130a-3p was elevated in cerebral AS patients and provided a possible method to predict cerebrovascular events (162). The different expressions of miR-130a-3p in AS period indicated the potential ability in AS diagnosis. However, we suppose that the signaling pathways of miR-130a-3p that implicate AS need to be discovered.

**Figure 2:**
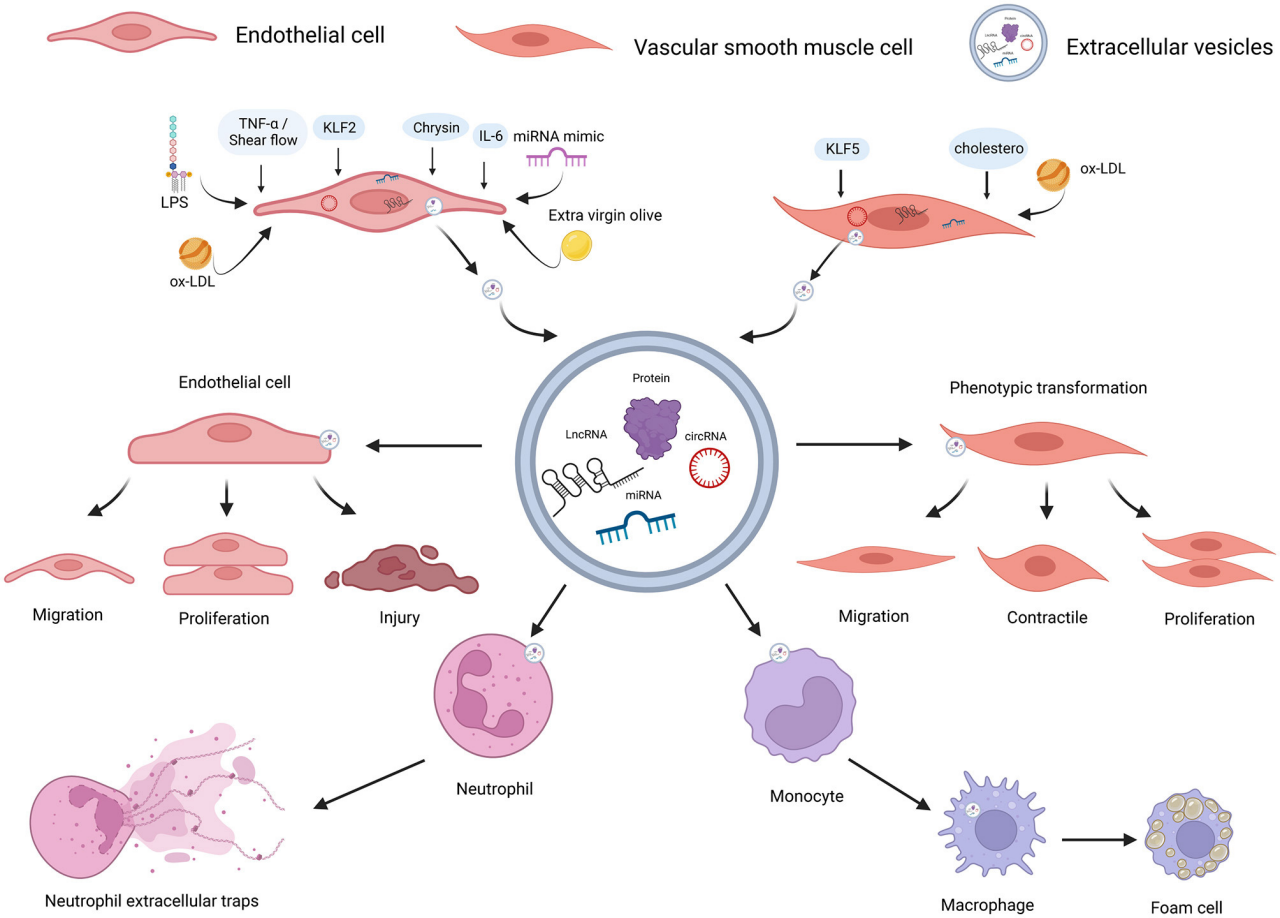
ncRNAs encapsulated in EC-EVs and VSMC-EVs regulate the AS process. Various stimuli stimulate the release of EVs and ncRNAs encapsulated in EC-EVs, and VSMC-EVs are assimilated by different recipient cells, resulting in AS inhibition or promotion. ncRNAs (miR-155-5p, miR-505-3p, miR-19b-3p, miR-4306, miR-92a-3p, MALAT1, ZEB1-AS1, CLDN10-AS1, LINC01005, and hsa_circ_0086296) encapsulated in EVs derived from ECs induced by different stimuli (LPS, extra-virgin-olive, ox-LDL, IL-6, TNF-α, shear flows, and chrysin) were linked to AS via enhancing ECs and VSMCs phenotype switching, promoting M1 macrophages activation, M2 macrophage polarization, NETs formation, lipid formation of macrophages, and endothelial injuries; miR-143-3p and miR-145-5p encapsulated in EVs derived from ECs stimulated by KLF2-transduced and shear-stress induced an atheroprotective VSMC phenotype; ncRNAs (miR-143-3p, miR-222-3p, miR-155-5p, and hsa_circ_0001445) encapsulated in EVs derived from VSMCs induced by stimuli (including hypercholesterolemia, KLF5, and ox-LD) were linked to AS by reducing expressions and enhancing endothelial injuries. ncRNAs, non-coding RNAs; VSMCs, vascular smooth muscle cells; ECs, endothelial cells; EVs, extracellular vesicles; AS, atherosclerosis; LPS, lipopolysaccharide; ox-LDL, oxidized low-density lipoprotein; IL-6, interleukin-6; TNF-α, tumor necrosis factor-a; NETs, neutrophil extracellular traps; KLF2, Krüppel-like factor 2; KLF5, Krüppel-like factor 5. Created with BioRender.com.

We have discussed the protective effect of miR-143-3p and the versatility of miR-222-3p, miR-155-5p, and miR-130a-3p in the process of AS. On the whole, versatile distributions between EVs and intracellular location probably perform profound effects on cellular metabolism, which implicated a variety of signaling pathways (Figure 2). Table 3 and Supplementary Table 3 indicate several miRNA-encapsulated VSMC-EVs that regulate the AS process.

**Table 3:** miRNAs encapsulated in VSMC-EVs regulate in the process of AS.

**miRNA**	**EVs origin**	**Stimulation**	**Expression**	**Function**	**References**
miR-143-3p	HCASMC	Hypercholesterolemia	Down	Cardiovascular biomarker	(140)
miR-222-3p	HCASMC	Hypercholesterolemia	Down	Cardiovascular biomarker	(140)
miR-155-5p	HASMC	KLF5	Up	Promoted as	(150)
miR-24-3p	HCASMC	Atherogenic lipoprotein	Down	Further researched required	(159)
miR-29b-3p	HCASMC	Atherogenic lipoprotein	Down	Further researched required	(159)
miR-130a-3p	HCASMC	Atherogenic lipoprotein	Down	Further researched required	(159)
miR143-3p	HCASMC	Atherogenic lipoprotein	Down	Further researched required	(159)
miR-222-3p	HCASMC	Atherogenic lipoprotein	Down	Further researched required	(159)

EVs, extracellular vesicles; HCASMC, human coronary artery smooth muscle cell; HASMC, human artery smooth muscle cell; KLF5, Krüppel-like factor 5; AS, atherosclerosis.

### 3.2. miRNAs encapsulated in VSMC-EVs regulate the process of VC

In VC, increased miR-92b-3p expression in both VSMCs and exosomes downregulated KLF4 and Runx2 expression in rat VSMCs (163). Similarly, Wang et al. (164) found that miR-92b-3p inhibited hypoxia-induced proliferation, migration, and phenotype switch of VSMCs.

MiR-204/miR-211 encapsulated in exosomes from melatonin-treated VSMCs attenuated VC by targeting bone morphogenetic protein-2 (BMP2) *in vitro* and in a 5/6 nephrectomy in C57BL/6 mice model (165). Indeed, miR-204 deficiency elevated valvular calcification activity and was independently associated with coronary artery calcification (CAC) and CVD in patients with type 2 diabetes mellitus (T2DM) (130, 131, 166–171). Correspondingly, the overexpression of miR-204 alleviated the osteoblastic differentiation of VSMCs *in vitro* and in female mice by regulating Runx2 (172, 173). In uremic rats, the expression of miR-29b increased, and the expressions of miR-133b and miR-211 decreased in calcifying conditions (107). Moreover, the expressions of miR-31, miR-106a, miR-148a, miR-204, miR-211, and miR-424 were lower in the aortic stenosis group than in the controls, whereas the levels of miR-30c were higher than in the controls (129). In conclusion, miR-92b-3p encapsulated in VSMC-EVs accelerated VC, while miR-204/miR-211 encapsulated in VSMC-EVs exhibited an anti-calcification function.

**Figure 3:**
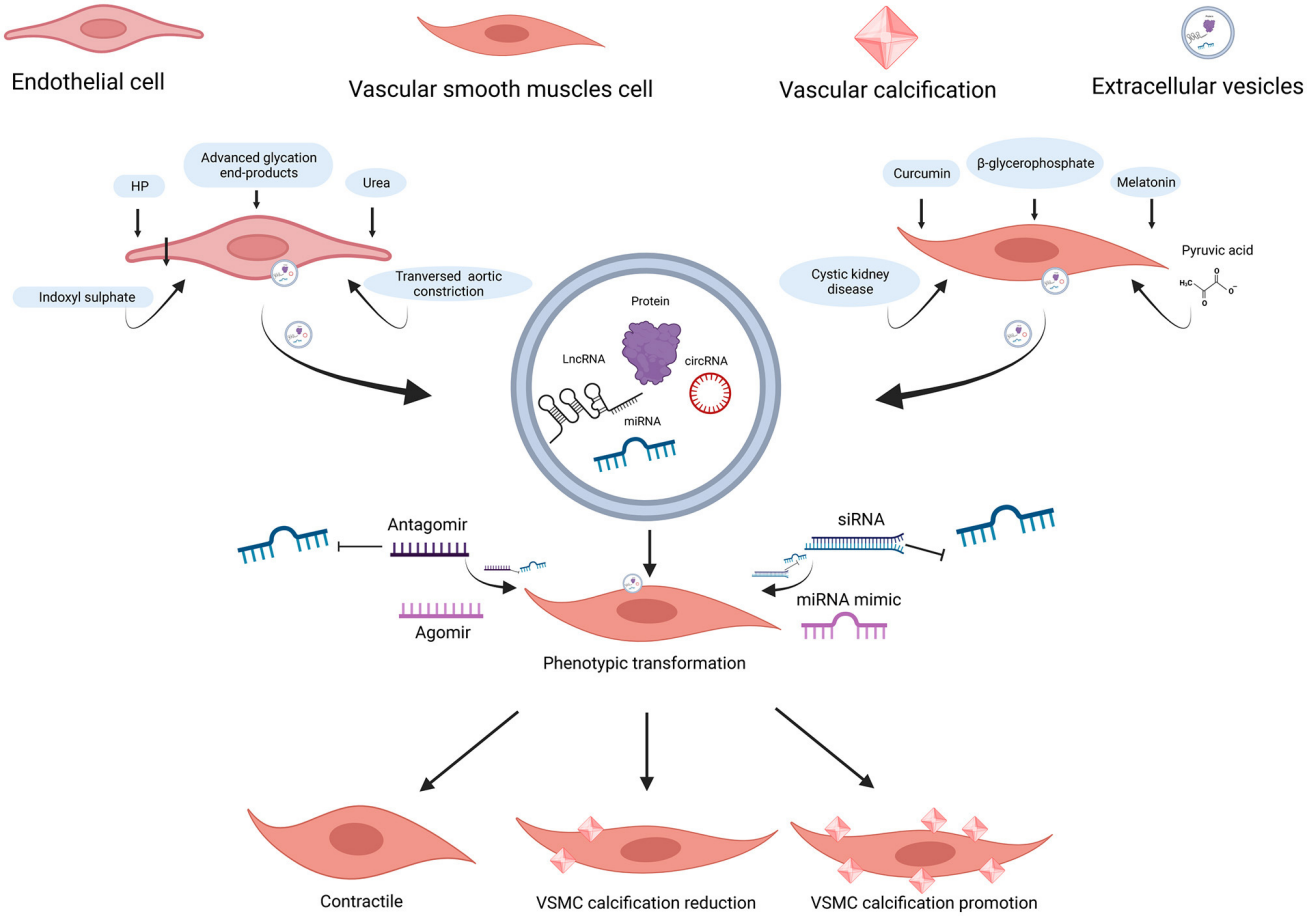
ncRNAs encapsulated in EC-EVs and VSMC-EVs regulate the VC process. Various stimuli stimulate the release of EVs and ncRNAs encapsulated in EC-EVs, and VSMC-EVs are assimilated by VSMCs, resulting in VC inhibition or promotion. miR-670-3p, miR-221-3p, and miR-222-3p encapsulated in EVs derived from ECs induced by stimuli (HP, uremic toxins, transverse aortic constriction, and advanced glycation end-products) promoted VC, while miR-143-3p, miR-145-5p, miR-29b-3p, and miR-126-5p inhibited VC; miR-92b-3p, miR-204-5p, and miR-211-5p encapsulated in EVs derived from VSMCs induced by stimuli (curcumin and melatonin), inhibited VC; the profiles of miRNAs encapsulated in EVs derived from VSMCs (β-GP and pyruvic acid-induced MOVAS-1 and cystic kidney disease rats VSMCs) have been detected. ncRNAs, non-coding RNAs; EVs, extracellular vesicles; VSMCs, vascular smooth muscle cells; ECs, endothelial cells; VC, vascular calcification; HP, high phosphate; β-GP, β-glycerophosphate. Created with BioRender.com.

Pan et al. found that 987 miRNAs were significantly upregulated, while 92 miRNAs were downregulated in exosomes from MOVAS-1 cells stimulated with β-glycerophosphate (β-GP) and pyruvic acid (174). Chaturvedi et al. (175) concluded that 14 miRNAs increased and 19 miRNAs decreased in MVs isolated from calcifying VSMCs obtained from descending thoracic aorta in three CKD rats. It is no coincidence that both research studies shared several common miRNAs: 7 miRNAs were up-regulated (miR-92b-5p, miR-204-3p, miR-296-3p, miR-494-3p, miR-667-5p, miR-702-5p, and miR-770-3p) while 6 miRNAs were down-regulated (let-7b-5p, miR-19b-3p, miR-24-3p, miR-30e-5p, miR-99b, and miR-199a-5p). Especially, miR-99b and miR-24-3p were decreased in VC condition in previous studies (104, 159). It also suggested that Runx2 was controlled by miR-30e, and the downregulation of miR-30e indicated osteoblast differentiation regulation (175). These findings facilitate the understanding of the effect of upregulation and downregulation of miRNAs in VSMC-EVs and VC, providing a bright prospect in the investigation of VC (Figure 3). Table 4 and Supplementary Table 4 describe several miRNAs that encapsulated VSMC-EVs that were regulated during the process of VC.

**Table 4:** miRNAs encapsulated in VSMC-EVs regulate in the process of VC.

**miRNA**	**EVs origin**	**Stimulation**	**Expression**	**Function**	**References**
miR-92b-3p	Rat VSMC	Curcumin	Up	Inhibited VC	(163)
miR-204-5p	Calcifying HVSMC	Melatonin	Up	Inhibited VC	(165)
miR-211-3p	Calcifying HVSMC	Melatonin	Up	Inhibited VC	(165)
miR-19b-3p	MOVAS-1	β-GP and pyruvic acid	Up	Further researched required	(174)
miR-19b-3p	Descending thoracic aorta	Cystic kidney disease	Up	Further researched required	(175)
miR-92b-5p	MOVAS-1	β-GP and pyruvic acid	Up	Further researched required	(174)
miR-92b-5p	Descending thoracic aorta	Cystic kidney disease	Up	Further researched required	(175)
miR-24-3p	Descending thoracic aorta	Cystic kidney disease	Down	Further researched required	(175)

EVs, extracellular vesicles; VSMC, vascular smooth muscle cell; HVSMC, human vascular smooth muscle cell; VC, vascular calcification; β-GP, β-glycerophosphate.

## 4. The emerging role of lncRNAs encapsulated in EVs in AS and VC processes

LncRNAs, another type of non-coding RNA larger than 200 base pairs, have significant enrichment in the nucleus compared with mRNAs, emerging as crucial regulators of tissue physiology and pathology mechanism (176–179). Many lncRNAs modulated gene expression in neuronal disorders and cancer (180). In turn, therapeutic targeting of lncRNAs provided a promising approach to the treatment of multiple diseases (181). Furthermore, lncRNA encapsulated in EVs represented an approach to utilizing lncRNAs in disease diagnostics and therapy (182). Here, we aimed to review studies related to lncRNAs encapsulated in EV in the process of AS and VC.

### 4.1. lncRNAs encapsulated in EVs involved in the process of AS

Indeed, different lncRNAs involved in AS showed opposite effects, such as lncRNA RNCR3 has an atheroprotective effect while lncRNA MIAT has a proinflammatory effect on VSMCs (183, 184). Moreover, lncRNAs encapsulated in EVs were also demonstrated to be tightly associated with AS (185). In mesenchymal stem cells (MSCs)-EVs, lncRNA FENDRR induced by ox-LDL alleviated HUVECs injury and AS by binding with miR-28 (186); knockdown of LOC100129516 alleviated the progression of AS (187). Encapsulated in ox-LDL-EVs from THP-1 cells, lncRNA GAS5 enhanced the apoptosis of HUVECs and HAECs and lncRNA LIPCAR-enhanced AS (188–190).

In ox-LDL-HUVEC-EVs, lncRNA MALAT1 promoted AS by inducing M2 macrophage polarization and NETs (191, 192); LncRNA ZEB1-AS1 promoted endothelial injuries in AS by competitively binding to miR-590-5p (193); LINC01005 promoted VSMC phenotype switch through miR-128-3p/KLF4 axis in the development of AS (194); lncRNA CLDN10-AS1 promoted endothelial injuries by sponging miR-186 (195).

In conclusion, lncRNAs encapsulated in EVs provided a new perspective on the intercellular communication of AS. The potential role of lncRNAs in the prevention and progress of AS still needs to be illuminated. Several lncRNAs encapsulated in EVs involved in the process of AS are summarized in Table 5.

**Table 5:** lncRNAs encapsulated in EVs involved in the process of AS.

**lncRNA**	**EVs origin**	**Stimulation**	**Expression**	**Function**	**References**
FENDRR	MSC	ox-LDL	Up	Inhibited as	(186)
LOC100129516	MSC	si-LOC100129516	Down	Promoted as	(187)
GAS5	THP-1	ox-LDL	Up	Promoted ec apoptosis	(188)
LIPCAR	THP-1	ox-LDL	Up	Promoted as	(189)
MALAT1	HUVEC	ox-LDL	Up	Promoted m2 macrophage polarization	(191)
MALAT1	HUVEC	ox-LDL	Up	Promoted as	(192)
ZEB1-AS1	HUVEC	ox-LDL	Up	Promoted as	(193)
LINC01005	HUVEC	ox-LDL	Up	Promoted as	(194)
CLDN10-AS1	HUVEC	ox-LDL	Up	Promoted ec injury	(195)

EVs, extracellular vesicles; MSC, mesenchymal stem cell; ox-LDL, oxidized low-density lipoprotein; AS, atherosclerosis; HUVEC, human umbilical vein endothelial cell; EC, endothelial cell.

### 4.2. lncRNAs involved in the process of VC

It has also been shown that the lncRNAs were new players regulating VC, providing therapeutic targets (196–198).

Bao et al. (199) revealed 728 different lncRNA expressions in HVSMCs under HP condition compared to the control group, in which 8 lncRNAs were the most potential lncRNAs in VC. Jeong et al. (200) discovered 100's of lncRNAs differentially expressed in rat VSMCs under HP condition, and Lrrc75a-as1 acted as an inhibitor of VC. LncRNA GAS5, as mentioned above (188, 189), promoted VC by modulating miR-26-5p (201). LncRNA SNHG1 alleviated high glucose-induced VSMC calcification by regulating basic helix–loop–helix family member e40 (Bhlhe40) (202). Similarly, lncRNA EPS inhibited VC in diabetic mice through the TGF-β/Wnt/β-catenin pathway (203). On the contrary, lncRNA LEF1-AS1 promoted VC (204). LncRNA-ES3 promoted VC by the modulation of miR-34c-5p and Bhlhe40 (205, 206).

Treated with HP, lncRNA H19/Runx2 axis promoted VC by the modulation of miR-103-3p (207–209). The modulation between LncRNA H19 and miR-138 was also discovered in the HP environment (210). Nevertheless, lncRNA H19 attenuated VC under ox-LDL and HP conditions in the development of AS (211).

However, there are no studies aimed to investigate the profile of lncRNAs encapsulated in EVs in the intercellular communication between ECs and VSMCs. This might be the reason that lncRNA expressions are relatively low in EVs so their transcripts cannot be analyzed in RNA-seq. More importantly, the lncRNA sequence–function relationship needs to be explored in the future (212). LncRNAs for the in-depth study often follow a specific physiologic or pathological state (213). The relationship between EVs and lncRNAs has the potential to advance our understanding of AS, VC, and other diseases.

## 5. The emerging role of circRNAs encapsulated in EVs in AS and VC processes

CircRNAs, generated from intronic lariats during colinear splicing and characterized by their covalently closed circular structure, showed tissue-specific and development stage-specific expression (214–216). Hansen et al. (217) first revealed that circRNAs were regarded as miRNAs sponges generally in gene regulation. Some endogenous circRNAs were highly abundant and evolutionarily conserved, providing potential implications for research and treatment applications (218). We have discussed miRNA encapsulated in EVs in AS and VC so far and subsequently aimed to summarize circRNA encapsulated in EVs in AS and VC.

### 5.1. circRNAs encapsulated in EVs involved in the process of AS

CircRNAs have been identified in the process of AS, such as circ_USP36, circ_0007478, circCHFR, and circ_0001879 (219–223). CircRNAs encapsulated in EVs were also involved in the process of AS (224). CircNPHP4 in monocyte-EVs regulated heterogeneous adhesion in coronary heart atherosclerotic disease by the modulation of miR-1231 (225). Hsa_circ_0001445 acted as a biomarker in CAD patients and was decreased in VSMCs-EVs on AS condition (226). Hsa_circ_0005699 was downregulated in the blood exosomes of patients with coronary heart disease and upregulated in ox-LDL-treated macrophage, providing candidate targets for the diagnosis of AS (227). Encapsulation in serum EVs of patients with unstable/vulnerable plaque AS, upregulated circRNA_0006896 plays a crucial role in carotid plaque destabilization by modulation of miR-1264 (228); circ_0043837 and circ_0001801 were independent predictive factors for artery atherosclerotic stroke (229). Hsa_circ_0086296 was also observed to be higher in AS patient serum EVs and ox-LDL-treated HUVECs, which promoted ECs injury and AS by the modulation of miR-576-3p (230). Although many circRNAs in AS have been revealed, circRNAs encapsulated in EVs during AS between intercellular communication will be focused, discussing issues and trends in studies in the future. Several circRNAs encapsulated in EVs involved in the process of AS are summarized in Table 6.

**Table 6:** circRNAs encapsulated in EVs involved in the process of AS.

**circRNA**	**EVs origin**	**Stimulation**	**Expression**	**Function**	**References**
CircNPHP4	Monocyte	Coronary heart atherosclerotic disease	Up	Biomarker for as	(225)
hsa_circ_0001445	HCASMC	Atherogenic lipoprotein	Down	Biomarker for cad	(226)
hsa_circ_0005699	Serum	Coronary heart disease	Down	Biomarker for as	(227)
circ_0006896	Serum	Unstable plaque as	Up	Linked to plaque destabilization	(228)
circ_0043837	Serum	Large artery atherosclerotic stroke	Down	Biomaker for stroke in as	(229)
circ_ 0001801	Serum	Large artery atherosclerotic stroke	Down	Biomaker for stroke in as	(229)
hsa_circ_0086296	HUVEC and serum	ox-LDL or AS	Up	Promoted as	(230)

EVs, extracellular vesicles; HCASMC, human coronary artery smooth muscle cell; AS, atherosclerosis; CAD, coronary artery disease; HUVEC, human umbilical vein endothelial cell; ox-LDL, oxidized low-density lipoprotein.

### 5.2. circRNAs involved in the process of VC

Ryu *et al*. discovered that circSamd4a reduced VC by modulating miR-125a-3p and miR-483-5p; circSmoc1-2 reduced VC by the modulation of miR-874-3p (231, 232). Moreover, CircSamd4a could act as a biomarker for the diagnosis of VC (233). In contrast, circRNA CDR1 promoted VC by sponging miR-7-5p (234). Similarly, hsa_circRNA_0008028-modulated miR-182-5p and promoted high glucose-induced VC (235). Notably, there were few articles concentrated on circRNAs participating in VC. Further studies are essential to elucidate the emerging role of circRNAs in the process of VC.

## 6. Discussion

In this review, we have summarized almost all miRNAs encapsulated in EVs secreted by ECs and VSMCs in the process of AS and VC. We concluded on the function of different miRNAs encapsulated in EVs which are effective to inhibit or aggravate AS and VC, depending on the stimuli and distributions. In addition, miRNAs encapsulated in EVs were expected to be indicators in the diagnosis and prognosis of cardiovascular diseases. There is still a long way to go. Different methods (direct electroporation, cell transfection, and chemical transfection) were developed to vehicle therapeutic molecules through EVs (236). Nevertheless, limitations, such as EV disruption and aggregation, still exist. The application of the atheroprotective miRNAs encapsulated in EVs in the treatment is still needed to be further investigated. In addition to elucidating the versatility of miRNAs in different signaling pathways, the complicated and subtle effects in the animal model should be illustrated.

Moreover, lncRNAs and circRNAs are expressed differentially during AS and VC. We have discussed the current understanding of lncRNAs and circRNAs in AS and VC. We have noticed the potential prospect of lncRNAs and circRNAs in the prevention, diagnosis, and treatment of AS/VC.

In conclusion, the versatile non-coding RNAs encapsulated in EVs provide a novel perspective on the biogenesis mechanisms of AS and VC.

## Author contributions

ZH and XX: conceptualization and supervision. FY and YD: writing—original draft preparation. HH, CL, and ZH: writing—review and editing. CL, HH, ZH, and XX: visualization. FY, CL, and ZH: reversion of the manuscript. ZH: project administration and funding acquisition. All authors have read and agreed to the published version of the manuscript.
