# Supplementary material for: Extracellular vesicles in atherosclerosis and vascular calcification: the versatile non-coding RNAs from endothelial cells and vascular smooth muscle cells
Source: Front Med (Lausanne). 2023 Jul 4;10:1193660. doi: 10.3389/fmed.2023.1193660 (PMC10352799; doi:10.3389/fmed.2023.1193660)
Supplement: Supplementary file 1 [file Data_Sheet_1.doc]

Supplementary Table 1. All miRNAs encapsulated in EC-EVs regulate the process of AS.

| miRNA | species | IDs | previous IDs | EV derived from | stimulant | expression | nucleotide sequence | function | reference |
| --- | --- | --- | --- | --- | --- | --- | --- | --- | --- |
| miR-27a-3p | human | hsa-miR-27a-3p | hsa-miR-27a | HUVEC | LPS | up | >hsa-miR-27a-3p MIMAT0000084 UUCACAGUGGCUAAGUUCCGC | various aspects of atherosclerotic lesion formation | 51 |
| miR-365b-3p | human | hsa-miR-365b-3p | hsa-miR-365b | HUVEC | LPS | up | >hsa-miR-365b-3p MIMAT0022834 UAAUGCCCCUAAAAAUCCUUAU | various aspects of atherosclerotic lesion formation | 51 |
| miR-126-5p | human | hsa-miR-126-5p | hsa-miR-126* | HUVEC | LPS | up | >hsa-miR-126-5p MIMAT0000444 CAUUAUUACUUUUGGUACGCG | various aspects of atherosclerotic lesion formation | 51 |
| miR-155-5p | human | hsa-miR-155-5p | hsa-miR-155 | HUVEC | LPS | up | >hsa-miR-155-5p MIMAT0000646 UUAAUGCUAAUCGUGAUAGGGGUU | various aspects of atherosclerotic lesion formation | 51 |
| miR-92a-3p | human | hsa-miR-92a-3p | hsa-miR-92/hsa-miR-92a | HUVEC | LPS | down | >hsa-miR-92a-3p MIMAT0000092 UAUUGCACUUGUCCCGGCCUGU | various aspects of atherosclerotic lesion formation | 51 |
| let-7b-5p | human | hsa-let-7b-5p | hsa-let-7b | HUVEC | LPS | down | >hsa-let-7b-5p MIMAT0000063 UGAGGUAGUAGGUUGUGUGGUU | various aspects of atherosclerotic lesion formation | 51 |
| miR-10a-5p | human | hsa-miR-10a-5p | hsa-miR-10a | HUVEC | LPS | down | >hsa-miR-10a-5p MIMAT0000253 UACCCUGUAGAUCCGAAUUUGUG | various aspects of atherosclerotic lesion formation | 51 |
| miR-10b-5p | human | hsa-miR-10b-5p | hsa-miR-10b | HUVEC | LPS | down | >hsa-miR-10b-5p MIMAT0000254 UACCCUGUAGAACCGAAUUUGUG | various aspects of atherosclerotic lesion formation | 51 |
| miR-21-3p | human | hsa-miR-21-3p | hsa-miR-21* | HUVEC | LPS | down | >hsa-miR-21-3p MIMAT0004494 CAACACCAGUCGAUGGGCUGU | various aspects of atherosclerotic lesion formation | 51 |
| miR-30a-5p | human | hsa-miR-30a-5p | hsa-miR-30a | HUVEC | LPS | down | >hsa-miR-30a-5p MIMAT0000087 UGUAAACAUCCUCGACUGGAAG | various aspects of atherosclerotic lesion formation | 51 |
| miR-125a-3p | human | hsa-miR-125a-3p | \ | HUVEC | LPS | down | >hsa-miR-125a-3p MIMAT0004602 ACAGGUGAGGUUCUUGGGAGCC | various aspects of atherosclerotic lesion formation | 51 |
| miR-143-3p | human | hsa-miR-143-3p | hsa-miR-143 | HUVEC | LPS | down | >hsa-miR-143-3p MIMAT0000435 UGAGAUGAAGCACUGUAGCUC | various aspects of atherosclerotic lesion formation | 51 |
| miR-181a-2-3p | human | hsa-miR-181a-2-3p | hsa- miR-181a-2* | HUVEC | LPS | down | >hsa-miR-181a-2-3p MIMAT0004558 ACCACUGACCGUUGACUGUACC | various aspects of atherosclerotic lesion formation | 51 |
| miR-216a-5p | human | hsa-miR-216a-5p | hsa-miR-216/hsa-miR-216a | HUVEC | extra-virgin-olive | up | >hsa-miR-216a-5p MIMAT0000273 UAAUCUCAGCUGGCAACUGUGA | closely linked to the development of AS | 52 |
| miR-31-3p | human | hsa-miR-31-3p | hsa-miR-31* | HUVEC | extra-virgin-olive | up | >hsa-miR-31-3p MIMAT0004504 UGCUAUGCCAACAUAUUGCCAU | closely linked to the development of AS | 52 |
| miR-20a-5p | human | hsa-miR-20a-5p | hsa-miR-20/hsa-miR-20a | HUVEC | extra-virgin-olive | up | >hsa-miR-20a-5p MIMAT0000075 UAAAGUGCUUAUAGUGCAGGUAG | closely linked to the development of AS | 52 |
| miR-126-5p | human | hsa-miR-126-5p | hsa-miR-126* | HUVEC | extra-virgin-olive | up | >hsa-miR-126-5p MIMAT0000444 CAUUAUUACUUUUGGUACGCG | closely linked to the development of AS | 52 |
| hsa-let-7g-5p | human | hsa-let-7g-5p | hsa-let-7g | HUVEC | extra-virgin-olive | up | >hsa-let-7g-5p MIMAT0000414 UGAGGUAGUAGUUUGUACAGUU | closely linked to the development of AS | 52 |
| hsa-miR-1271-5p | human | hsa-miR-1271-5p | hsa-miR-1271 | HUVEC | extra-virgin-olive | up | >hsa-miR-1271-5p MIMAT0005796 CUUGGCACCUAGCAAGCACUCA | closely linked to the development of AS | 52 |
| hsa-miR-148b-3p | human | hsa-miR-148b-3p | hsa-miR-148b | HUVEC | extra-virgin-olive | up | >hsa-miR-148b-3p MIMAT0000759 UCAGUGCAUCACAGAACUUUGU | closely linked to the development of AS | 52 |
| hsa-miR-195-5p | human | hsa-miR-195-5p | hsa-miR-195 | HUVEC | extra-virgin-olive | up | >hsa-miR-195-5p MIMAT0000461 UAGCAGCACAGAAAUAUUGGC | closely linked to the development of AS | 52 |
| hsa-miR-216a-3p | human | hsa-miR-216a-3p | \ | HUVEC | extra-virgin-olive | up | >hsa-miR-216a-3p MIMAT0022844 UCACAGUGGUCUCUGGGAUUAU | closely linked to the development of AS | 52 |
| hsa-miR-27b-3p | human | hsa-miR-27b-3p | hsa-miR-27b | HUVEC | extra-virgin-olive | up | >hsa-miR-27b-3p MIMAT0000419 UUCACAGUGGCUAAGUUCUGC | closely linked to the development of AS | 52 |
| hsa-miR-345-5p | human | hsa-miR-345-5p | hsa-miR-345 | HUVEC | extra-virgin-olive | up | >hsa-miR-345-5p MIMAT0000772 GCUGACUCCUAGUCCAGGGCUC | closely linked to the development of AS | 52 |
| hsa-miR-452-5p | human | hsa-miR-452-5p | hsa-miR-452 | HUVEC | extra-virgin-olive | up | >hsa-miR-452-5p MIMAT0001635 AACUGUUUGCAGAGGAAACUGA | closely linked to the development of AS | 52 |
| hsa-miR-652-3p | human | hsa-miR-652-3p | hsa-miR-652 | HUVEC | extra-virgin-olive | up | >hsa-miR-652-3p MIMAT0003322 AAUGGCGCCACUAGGGUUGUG | closely linked to the development of AS | 52 |
| hsa-miR-93-5p | human | hsa-miR-93-5p | hsa-miR-93 | HUVEC | extra-virgin-olive | up | >hsa-miR-93-5p MIMAT0000093 CAAAGUGCUGUUCGUGCAGGUAG | closely linked to the development of AS | 52 |
| hsa-miR-106b-5p | human | hsa-miR-106b-5p | hsa-miR-106b | HUVEC | extra-virgin-olive | up | >hsa-miR-106b-5p MIMAT0000680 UAAAGUGCUGACAGUGCAGAU | closely linked to the development of AS | 52 |
| hsa-miR-1307-5p | human | hsa-miR-1307-5p | \ | HUVEC | extra-virgin-olive | up | >hsa-miR-1307-5p MIMAT0022727 UCGACCGGACCUCGACCGGCU | closely linked to the development of AS | 52 |
| hsa-miR-16-5p | human | hsa-miR-16-5p | hsa-miR-16 | HUVEC | extra-virgin-olive | up | >hsa-miR-16-5p MIMAT0000069 UAGCAGCACGUAAAUAUUGGCG | closely linked to the development of AS | 52 |
| hsa-miR-19a-3p | human | hsa-miR-19a-3p | hsa-miR-19a | HUVEC | extra-virgin-olive | up | >hsa-miR-19a-3p MIMAT0000073 UGUGCAAAUCUAUGCAAAACUGA | closely linked to the development of AS | 52 |
| hsa-miR-301b-3p | human | hsa-miR-301b-3p | hsa-miR-301b | HUVEC | extra-virgin-olive | up | >hsa-miR-301b-3p MIMAT0004958 CAGUGCAAUGAUAUUGUCAAAGC | closely linked to the development of AS | 52 |
| hsa-miR-31-5p | human | hsa-miR-31-5p | hsa-miR-31 | HUVEC | extra-virgin-olive | up | >hsa-miR-31-5p MIMAT0000089 AGGCAAGAUGCUGGCAUAGCU | closely linked to the development of AS | 52 |
| hsa-miR-361-5p | human | hsa-miR-361-5p | hsa-miR-361 | HUVEC | extra-virgin-olive | up | >hsa-miR-361-5p MIMAT0000703 UUAUCAGAAUCUCCAGGGGUAC | closely linked to the development of AS | 52 |
| hsa-miR-574-3p | human | hsa-miR-574-3p | hsa-miR-574 | HUVEC | extra-virgin-olive | up | >hsa-miR-574-3p MIMAT0003239 CACGCUCAUGCACACACCCACA | closely linked to the development of AS | 52 |
| hsa-miR-671-5p | human | hsa-miR-671-5p | hsa-miR-671 | HUVEC | extra-virgin-olive | up | >hsa-miR-671-5p MIMAT0003880 AGGAAGCCCUGGAGGGGCUGGAG | closely linked to the development of AS | 52 |
| hsa-miR-125b-2-3p | human | hsa-miR-125b-2-3p | hsa-miR-125b-2* | HUVEC | extra-virgin-olive | up | >hsa-miR-125b-2-3p MIMAT0004603 UCACAAGUCAGGCUCUUGGGAC | closely linked to the development of AS | 52 |
| hsa-miR-132-3p | human | hsa-miR-132-3p | hsa-miR-132 | HUVEC | extra-virgin-olive | up | >hsa-miR-132-3p MIMAT0000426 UAACAGUCUACAGCCAUGGUCG | closely linked to the development of AS | 52 |
| hsa-miR-181b-5p | human | hsa-miR-181b-5p | hsa-miR-181b | HUVEC | extra-virgin-olive | up | >hsa-miR-181b-5p MIMAT0000257 AACAUUCAUUGCUGUCGGUGGGU | closely linked to the development of AS | 52 |
| hsa-miR-19b-3p | human | hsa-miR-19b-3p | hsa-miR-19b | HUVEC | extra-virgin-olive | up | >hsa-miR-19b-3p MIMAT0000074 UGUGCAAAUCCAUGCAAAACUGA | closely linked to the development of AS | 52 |
| hsa-miR-217 | human | hsa-miR-217-5p | \ | HUVEC | extra-virgin-olive | up | >hsa-miR-217-5p MIMAT0000274 UACUGCAUCAGGAACUGAUUGGA | closely linked to the development of AS | 52 |
| hsa-miR-30b-5p | human | hsa-miR-30b-5p | hsa-miR-30b | HUVEC | extra-virgin-olive | up | >hsa-miR-30b-5p MIMAT0000420 UGUAAACAUCCUACACUCAGCU | closely linked to the development of AS | 52 |
| hsa-miR-3200-3p | human | hsa-miR-3200-3p | hsa-miR-3200 | HUVEC | extra-virgin-olive | up | >hsa-miR-3200-3p MIMAT0015085 CACCUUGCGCUACUCAGGUCUG | closely linked to the development of AS | 52 |
| hsa-miR-425-3p | human | hsa-miR-425-3p | hsa-miR-425* | HUVEC | extra-virgin-olive | up | >hsa-miR-425-3p MIMAT0001343 AUCGGGAAUGUCGUGUCCGCCC | closely linked to the development of AS | 52 |
| hsa-miR-582-3p | human | hsa-miR-582-3p | \ | HUVEC | extra-virgin-olive | up | >hsa-miR-582-3p MIMAT0004797 UAACUGGUUGAACAACUGAACC | closely linked to the development of AS | 52 |
| hsa-miR-874-3p | human | hsa-miR-874-3p | \ | HUVEC | extra-virgin-olive | up | >hsa-miR-874-3p MIMAT0004911 CUGCCCUGGCCCGAGGGACCGA | closely linked to the development of AS | 52 |
| hsa-miR-140-3p | human | hsa-miR-140-3p | \ | HUVEC | extra-virgin-olive | up | >hsa-miR-140-3p MIMAT0004597 UACCACAGGGUAGAACCACGG | closely linked to the development of AS | 52 |
| hsa-miR-181d-5p | human | hsa-miR-181d-5p | \ | HUVEC | extra-virgin-olive | up | >hsa-miR-181d-5p MIMAT0002821 AACAUUCAUUGUUGUCGGUGGGU | closely linked to the development of AS | 52 |
| hsa-miR-23a-3p | human | hsa-miR-23a-3p | hsa-miR-23a | HUVEC | extra-virgin-olive | up | >hsa-miR-23a-3p MIMAT0000078 AUCACAUUGCCAGGGAUUUCC | closely linked to the development of AS | 52 |
| hsa-miR-30c-5p | human | hsa-miR-30c-5p | hsa-miR-30c | HUVEC | extra-virgin-olive | up | >hsa-miR-30c-5p MIMAT0000244 UGUAAACAUCCUACACUCUCAGC | closely linked to the development of AS | 52 |
| hsa-miR-324-3p | human | hsa-miR-324-3p | \ | HUVEC | extra-virgin-olive | up | >hsa-miR-324-3p MIMAT0000762 CCCACUGCCCCAGGUGCUGCUGG | closely linked to the development of AS | 52 |
| hsa-miR-425-5p | human | hsa-miR-425-5p | hsa-miR-425 | HUVEC | extra-virgin-olive | up | >hsa-miR-425-5p MIMAT0003393 AAUGACACGAUCACUCCCGUUGA | closely linked to the development of AS | 52 |
| hsa-miR-641 | human | hsa-miR-641 | \ | HUVEC | extra-virgin-olive | up | >hsa-miR-641 MIMAT0003311 AAAGACAUAGGAUAGAGUCACCUC | closely linked to the development of AS | 52 |
| hsa-miR-887-3p | human | hsa-miR-887-3p | \ | HUVEC | extra-virgin-olive | up | >hsa-miR-887-3p MIMAT0004951 GUGAACGGGCGCCAUCCCGAGG | closely linked to the development of AS | 52 |
| hsa-miR-127-3p | human | hsa-miR-127-3p | hsa-miR-127 | HUVEC | extra-virgin-olive | down | >hsa-miR-127-3p MIMAT0000446 UCGGAUCCGUCUGAGCUUGGCU | closely linked to the development of AS | 52 |
| hsa-miR-130b-5p | human | hsa-miR-130b-5p | hsa-miR-130b* | HUVEC | extra-virgin-olive | down | >hsa-miR-130b-5p MIMAT0004680 ACUCUUUCCCUGUUGCACUAC | closely linked to the development of AS | 52 |
| hsa-miR-183-5p | human | hsa-miR-183-5p | hsa-miR-183 | HUVEC | extra-virgin-olive | down | >hsa-miR-183-5p MIMAT0000261 UAUGGCACUGGUAGAAUUCACU | closely linked to the development of AS | 52 |
| hsa-miR-204-3p | human | hsa-miR-204-3p | \ | HUVEC | extra-virgin-olive | down | >hsa-miR-204-3p MIMAT0022693 GCUGGGAAGGCAAAGGGACGU | closely linked to the development of AS | 52 |
| hsa-miR-222-3p | human | hsa-miR-222-3p | hsa-miR-222 | HUVEC | extra-virgin-olive | down | >hsa-miR-222-3p MIMAT0000279 AGCUACAUCUGGCUACUGGGU | closely linked to the development of AS | 52 |
| hsa-miR-409-3p | human | hsa-miR-409-3p | \ | HUVEC | extra-virgin-olive | down | >hsa-miR-409-5p MIMAT0001638 AGGUUACCCGAGCAACUUUGCAU | closely linked to the development of AS | 52 |
| hsa-miR-424-3p | human | hsa-miR-424-3p | hsa-miR-424* | HUVEC | extra-virgin-olive | down | >hsa-miR-424-3p MIMAT0004749 CAAAACGUGAGGCGCUGCUAU | closely linked to the development of AS | 52 |
| hsa-miR-485-3p | human | hsa-miR-485-3p | \ | HUVEC | extra-virgin-olive | down | >hsa-miR-485-3p MIMAT0002176 GUCAUACACGGCUCUCCUCUCU | closely linked to the development of AS | 52 |
| hsa-miR-543 | human | hsa-miR-543 | \ | HUVEC | extra-virgin-olive | down | >hsa-miR-543 MIMAT0004954 AAACAUUCGCGGUGCACUUCUU | closely linked to the development of AS | 52 |
| hsa-miR-671-3p | human | hsa-miR-671-3p | \ | HUVEC | extra-virgin-olive | down | >hsa-miR-671-3p MIMAT0004819 UCCGGUUCUCAGGGCUCCACC | closely linked to the development of AS | 52 |
| hsa-miR-128-3p | human | hsa-miR-128-3p | hsa-miR-128a | HUVEC | extra-virgin-olive | down | >hsa-miR-128-3p MIMAT0000424 UCACAGUGAACCGGUCUCUUU | closely linked to the development of AS | 52 |
| hsa-miR-1908-5p | human | hsa-miR-1908-5p | \ | HUVEC | extra-virgin-olive | down | >hsa-miR-1908-5p MIMAT0007881 CGGCGGGGACGGCGAUUGGUC | closely linked to the development of AS | 52 |
| hsa-miR-204-5p | human | hsa-miR-204-5p | hsa-miR-204 | HUVEC | extra-virgin-olive | down | >hsa-miR-204-5p MIMAT0000265 UUCCCUUUGUCAUCCUAUGCCU | closely linked to the development of AS | 52 |
| hsa-miR-323a-5p | human | hsa-miR-323a-5p | \ | HUVEC | extra-virgin-olive | down | >hsa-miR-323a-5p MIMAT0004696 AGGUGGUCCGUGGCGCGUUCGC | closely linked to the development of AS | 52 |
| hsa-miR-411-3p | human | hsa-miR-411-3p | hsa-miR-411* | HUVEC | extra-virgin-olive | down | >hsa-miR-411-3p MIMAT0004813 UAUGUAACACGGUCCACUAACC | closely linked to the development of AS | 52 |
| hsa-miR-4488 | human | hsa-miR-4488 | \ | HUVEC | extra-virgin-olive | down | >hsa-miR-4488 MIMAT0019022 AGGGGGCGGGCUCCGGCG | closely linked to the development of AS | 52 |
| hsa-miR-485-5p | human | hsa-miR-485-5p | \ | HUVEC | extra-virgin-olive | down | >hsa-miR-485-5p MIMAT0002175 AGAGGCUGGCCGUGAUGAAUUC | closely linked to the development of AS | 52 |
| hsa-miR-615-3p | human | hsa-miR-615-3p | hsa-miR-615 | HUVEC | extra-virgin-olive | down | >hsa-miR-615-3p MIMAT0003283 UCCGAGCCUGGGUCUCCCUCUU | closely linked to the development of AS | 52 |
| miR-204-5p | human | hsa-miR-204-5p | hsa-miR-204 | HUVEC | extra-virgin-olive | down | >hsa-miR-204-5p MIMAT0000265 UUCCCUUUGUCAUCCUAUGCCU | closely linked to the development of AS | 52 |
| miR-155 | human | hsa-miR-155-5p | hsa-miR-155 | HUVEC | ox-LDL | up | >hsa-miR-155-5p MIMAT0000646 UUAAUGCUAAUCGUGAUAGGGGUU | promoted AS by enhancing monocyte activation and downregulated by KLF2 | 54 |
| miR-155 | mice | mmu-miR-155-5p | mmu-miR-155 | mouse EC | high-fat diet | up | >mmu-miR-155-5p MIMAT0000165 UUAAUGCUAAUUGUGAUAGGGGU | promoted AS by enhancing monocyte activation and downregulated by KLF2 | 54 |
| miR-505 | human | hsa-miR-505-3p | hsa-miR-505 | HUVEC | ox-LDL | up | >hsa-miR-505-3p MIMAT0002876 CGUCAACACUUGCUGGUUUCCU | promoted AS by inducing NET formation | 55 |
| miR-19b | human | hsa-miR-19b-3p | hsa-miR-19b | HUVEC | miR-19b mimic | up | >hsa-miR-19b-3p MIMAT0000074 UGUGCAAAUCCAUGCAAAACUGA | promoted AS by downregulating TGF-βRII | 56 |
| miR-19b | mice | mmu-miR-19b-3p | mmu-miR-19b | \ | miR-19b mimic | up | >mmu-miR-19b-3p MIMAT0000513 UGUGCAAAUCCAUGCAAAACUGA | promoted AS by downregulating TGF-βRII | 56 |
| miR-4306 | human | hsa-miR-4306 | \ | HCAEC | ox-LDL | up | >hsa-miR-4306 MIMAT0016858 UGGAGAGAAAGGCAGUA | promoted AS by upregulating Akt/NF-κB | 57 |
| miR-92a | human | hsa-miR-92a-3p | hsa-miR-92/hsa-miR-92a | HUVEC | TNF-α or shear flows | up | >hsa-miR-92a-3p MIMAT0000092 UAUUGCACUUGUCCCGGCCUGU | promoted AS by downregulating KLF4 | 58 |
| miR-92a-3p | human | hsa-miR-92a-3p | hsa-miR-92/hsa-miR-92a | HCAEC | oxLDL and IL-6 | up | >hsa-miR-92a-3p MIMAT0000092 UAUUGCACUUGUCCCGGCCUGU | promoted angiogenesis by downregulating THBS1 | 59 |
| miR-92a | human | hsa-miR-92a-3p | hsa-miR-92/hsa-miR-92a | HCAEC | chrysin | down | >hsa-miR-92a-3p MIMAT0000092 UAUUGCACUUGUCCCGGCCUGU | promoted AS by inhibiting KLF2 | 60 |
| miR-143 | human | hsa-miR-143-3p | hsa-miR-143 | HUVEC | KLF2-transduced or shear-stress-stimulated | up | >hsa-miR-143-3p MIMAT0000435 UGAGAUGAAGCACUGUAGCUC | inhibited AS by inducing an atheroprotective HASMC phenotype | 61 |
| miR-143 | mice | mmu-miR-143-3p | mmu-miR-143 | mouse EC | KLF2-transduced | up | >mmu-miR-143-3p MIMAT0000247 UGAGAUGAAGCACUGUAGCUC | inhibited AS by inducing an atheroprotective HASMC phenotype | 61 |
| miR-145 | human | hsa-miR-145-5p | hsa-miR-145 | HUVEC | KLF2-transduced or shear-stress-stimulated | up | >hsa-miR-145-5p MIMAT0000437 GUCCAGUUUUCCCAGGAAUCCCU | inhibited AS by inducing an atheroprotective HASMC phenotype | 61 |
| miR-145 | mice | mmu-miR-145a-5p | mmu-miR-145/mmu-miR-145-5p | mouse EC | KLF2-transduced | up | >mmu-miR-145a-5p MIMAT0000157 GUCCAGUUUUCCCAGGAAUCCCU | inhibited AS by inducing an atheroprotective HASMC phenotype | 61 |

Supplementary Table 2. All miRNAs encapsulated in EC-EVs regulate the process of VC.

| miRNA | species | IDs | previous IDs | EV derived from | stimulant | expression | nucleotide sequence | function | reference |
| --- | --- | --- | --- | --- | --- | --- | --- | --- | --- |
| miR-670-3p | mice | mmu-miR-670-3p | mmu-miR-670* | mice EC | HP | up | >mmu-miR-670-3p MIMAT0017242 UUUCCUCAUAUCCAUUCAGGAGUGU | promoted VC by targeting IGF-1 | 100 |
| miR‐221 | human | hsa-miR‐221-3p | hsa-miR‐221 | human EC | urea and indoxyl sulphate | up | >hsa-miR-221-3p MIMAT0000278 AGCUACAUUGUCUGCUGGGUUUC | pro-calcifying effect | 101 |
| miR‐221 | rat | rno-miR‐221-3p | rno-miR‐221 | \ | \ | \ | >rno-miR-221-3p MIMAT0000890 AGCUACAUUGUCUGCUGGGUUUC | \ | 101 |
| miR-222 | human | hsa-miR‐222-3p | hsa-miR‐222 | human EC | urea and indoxyl sulphate | up | >hsa-miR-222-3p MIMAT0000279 AGCUACAUCUGGCUACUGGGU | pro-calcifying effects with miR‐221-3p | 101 |
| miR-222 | rat | rno-miR‐222-3p | rno-miR‐222 | \ | \ | \ | >rno-miR-222-3p MIMAT0000891 AGCUACAUCUGGCUACUGGGU | \ | 101 |
| miR-126 | human | hsa-miR‐126-3p | hsa-miR‐126 | human EC | urea and indoxyl sulphate | up | >hsa-miR-126-3p MIMAT0000445 UCGUACCGUGAGUAAUAAUGCG | no pro-calcifying effect | 101 |
| miR-126 | rat | rno-miR‐126a-3p | rno-miR‐126 | \ | \ | \ | >rno-miR-126a-3p MIMAT0000832 UCGUACCGUGAGUAAUAAUGCG | \ | 101 |
| miR-143 | human | hsa-miR-143-3p | hsa-miR-143 | human EC | urea and indoxyl sulphate | down | >hsa-miR-143-3p MIMAT0000435 UGAGAUGAAGCACUGUAGCUC | inhibited VC | 101 |
| miR-143 | rat | rno-miR‐143-3p | rno-miR‐143 | \ | \ | \ | >rno-miR-143-3p MIMAT0000849 UGAGAUGAAGCACUGUAGCUCA | \ | 101 |
| miR-145 | human | hsa-miR-145-5p | hsa-miR-145 | human EC | urea and indoxyl sulphate | down | >hsa-miR-145-5p MIMAT0000437 GUCCAGUUUUCCCAGGAAUCCCU | inhibited VC | 101 |
| miR-145 | rat | rno-miR‐145-5p | rno-miR‐145 | \ | \ | \ | >rno-miR-145-5p MIMAT0000851 GUCCAGUUUUCCCAGGAAUCCCU | \ | 101 |
| miR-29b | mice | mmu-miR-29b-3p | mmu-miR-29b | mouse AEC | transverse aortic constriction | down | >mmu-miR-29b-3p MIMAT0000127 UAGCACCAUUUGAAAUCAGUGUU | inhibited vascular stiffness | 102 |
| miR-29b | human | hsa-miR-29b-3p | hsa-miR-29b | \ | \ | \ | >hsa-miR-29b-3p MIMAT0000100 UAGCACCAUUUGAAAUCAGUGUU | \ | \ |
| miR-29b | rat | rno-miR-29b-3p | rno-miR-29b | \ | \ | \ | >rno-miR-29b-3p MIMAT0000801 UAGCACCAUUUGAAAUCAGUGUU | \ | \ |
| miR-126-5p | human | hsa-miR-126-5p | hsa-miR-126* | HUVEC | advanced glycation end-products | up | >hsa-miR-126-5p MIMAT0000444 CAUUAUUACUUUUGGUACGCG | inhibited VC through smad1/5/9 signalling pathway | 103 |
| miR-3182 | human | hsa-miR-3182 | \ | HUVEC | HP | up | >hsa-miR-3182 MIMAT0015062 GCUUCUGUAGUGUAGUC | further researches required | 104 |
| miR-10a-5p | human | hsa-miR-10a-5p | hsa-miR-10a | HUVEC | HP | down | >hsa-miR-10a-5p MIMAT0000253 UACCCUGUAGAUCCGAAUUUGUG | further researches required | 104 |
| miR-10b-5p | human | hsa-miR-10b-5p | hsa-miR-10b | HUVEC | HP | down | >hsa-miR-10b-5p MIMAT0000254 UACCCUGUAGAACCGAAUUUGUG | further researches required | 104 |
| miR-30a-3p | human | hsa-miR-30a-3p | hsa-miR-30a* | HUVEC | HP | down | >hsa-miR-30a-3p MIMAT0000088 CUUUCAGUCGGAUGUUUGCAGC | further researches required | 104 |
| miR-30a-5p | human | hsa-miR-30a-5p | hsa-miR-30a | HUVEC | HP | down | >hsa-miR-30a-5p MIMAT0000087 UGUAAACAUCCUCGACUGGAAG | further researches required | 104 |
| miR-30a-5p | mice | mmu-miR-30a-5p | mmu-miR-30a | \ | \ | \ | >mmu-miR-30a-5p MIMAT0000128 UGUAAACAUCCUCGACUGGAAG | \ | \ |
| miR-30c-2-3p | human | hsa-miR-30c-2-3p | hsa-miR-30c-2* | HUVEC | HP | down | >hsa-miR-30c-2-3p MIMAT0004550 CUGGGAGAAGGCUGUUUACUCU | further researches required | 104 |
| miR-99b-5p | human | hsa-miR-99b-5p | hsa-miR-99b | HUVEC | HP | down | >hsa-miR-99b-5p MIMAT0000689 CACCCGUAGAACCGACCUUGCG | further researches required | 104 |
| miR-143-3p | human | hsa-miR-143-3p | hsa-miR-143 | HUVEC | HP | down | >hsa-miR-143-3p MIMAT0000435 UGAGAUGAAGCACUGUAGCUC | further researches required | 104 |
| miR-193b-5p | human | hsa-miR-193b-5p | hsa-miR-193b* | HUVEC | HP | down | >hsa-miR-193b-5p MIMAT0004767 CGGGGUUUUGAGGGCGAGAUGA | further researches required | 104 |
| miR-365a-5p | human | hsa-miR-365a-5p | hsa-miR-365a* | HUVEC | HP | down | >hsa-miR-365a-5p MIMAT0009199 AGGGACUUUUGGGGGCAGAUGUG | further researches required | 104 |
| miR-486-5p | human | hsa-miR-486-5p | hsa-miR-486 | HUVEC | HP | down | >hsa-miR-486-5p MIMAT0002177 UCCUGUACUGAGCUGCCCCGAG | further researches required | 104 |
| miR-941 | human | hsa-miR-941 | \ | HUVEC | HP | down | >hsa-miR-941 MIMAT0004984 CACCCGGCUGUGUGCACAUGUGC | further researches required | 104 |
| miR-7706 | human | hsa-miR-7706 | \ | HUVEC | HP | down | >hsa-miR-7706 MIMAT0030021 UGAAGCGCCUGUGCUCUGCCGAGA | further researches required | 104 |

Supplementary Table 3. All miRNAs encapsulated in VSMC-EVs regulate in the process of AS.

| miRNA | species | IDs | previous IDs | EV derived from | stimulant | expression | nucleotide sequence | function | reference |
| --- | --- | --- | --- | --- | --- | --- | --- | --- | --- |
| miR-143 | human | hsa-miR-143-3p | hsa-miR-143 | HCASMC | hypercholesterolemia | down | >hsa-miR-143-3p MIMAT0000435 UGAGAUGAAGCACUGUAGCUC | cardiovascular biomarker | 140 |
| miR-222 | human | hsa-miR‐222-3p | hsa-miR‐222 | HCASMC | hypercholesterolemia | down | >hsa-miR-222-3p MIMAT0000279 AGCUACAUCUGGCUACUGGGU | cardiovascular biomarker | 140 |
| miR-155 | human | hsa-miR-155-5p | hsa-miR-155 | HASMC | KLF5 | up | >hsa-miR-155-5p MIMAT0000646 UUAAUGCUAAUCGUGAUAGGGGUU | pro-atherosclerotic | 150 |
| miR-15b-5p | human | hsa-miR-15b-5p | hsa-miR-15b | HCASMC | atherogenic lipoprotein | down | >hsa-miR-15b-5p MIMAT0000417 UAGCAGCACAUCAUGGUUUACA | further researches required | 159 |
| miR-24-3p | human | hsa-miR-24-3p | hsa-miR-24 | HCASMC | atherogenic lipoprotein | down | >hsa-miR-24-3p MIMAT0000080 UGGCUCAGUUCAGCAGGAACAG | further researches required | 159 |
| miR-29b-3p | human | hsa-miR-29b-3p | hsa-miR-29b | HCASMC | atherogenic lipoprotein | down | >hsa-miR-29b-3p MIMAT0000100 UAGCACCAUUUGAAAUCAGUGUU | further researches required | 159 |
| miR-130a-3p | human | hsa-miR-130a-3p | hsa-miR-130a | HCASMC | atherogenic lipoprotein | down | >hsa-miR-130a-3p MIMAT0000425 CAGUGCAAUGUUAAAAGGGCAU | further researches required | 159 |
| miR-143-3p | human | hsa-miR-143-3p | hsa-miR-143 | HCASMC | atherogenic lipoprotein | down | >hsa-miR-143-3p MIMAT0000435 UGAGAUGAAGCACUGUAGCUC | further researches required | 159 |
| miR-146a-3p | human | hsa-miR-146a-3p | hsa-miR-146a* | HCASMC | atherogenic lipoprotein | down | >hsa-miR-146a-3p MIMAT0004608 CCUCUGAAAUUCAGUUCUUCAG | further researches required | 159 |
| miR-222-3p | human | hsa-miR‐222-3p | hsa-miR‐222 | HCASMC | atherogenic lipoprotein | down | >hsa-miR-222-3p MIMAT0000279 AGCUACAUCUGGCUACUGGGU | further researches required | 159 |
| miR-663a | human | hsa-miR-663a | hsa-miR-663 | HCASMC | atherogenic lipoprotein | down | >hsa-miR-663a MIMAT0003326 AGGCGGGGCGCCGCGGGACCGC | further researches required | 159 |

Supplementary Table 4. All miRNAs encapsulated in VSMC-EVs regulate in the process of VC.

| miRNA | species | IDs | previous IDs | EV derived from | stimulant | expression | nucleotide sequence | function | reference |
| --- | --- | --- | --- | --- | --- | --- | --- | --- | --- |
| miR-92b-3p | rat | rno-miR-92b-3p | rno-miR-92b | rat VSMCs | curcumin | up | >rno-miR-92b-3p MIMAT0005340 UAUUGCACUCGUCCCGGCCUCC | inhibited VC | 163 |
| miR-92b-3p | human | hsa-miR-92b-3p | hsa-miR-92b | \ | \ | \ | >hsa-miR-92b-3p MIMAT0003218 UAUUGCACUCGUCCCGGCCUCC | \ | \ |
| miR‐204 | human | hsa-miR-204-5p | hsa-miR-204 | calcifying HVSMCs | melatonin | up | >hsa-miR-204-5p MIMAT0000265 UUCCCUUUGUCAUCCUAUGCCU | inhibited VC | 165 |
| miR‐204 | mice | mmu-miR-204-5p | mmu-miR-204 | \ | \ | \ | >mmu-miR-204-5p MIMAT0000237 UUCCCUUUGUCAUCCUAUGCCU | \ | \ |
| miR‐211 | human | hsa-miR-211-5p | hsa-miR-211 | calcifying HVSMCs | melatonin | up | >hsa-miR-211-5p MIMAT0000268 UUCCCUUUGUCAUCCUUCGCCU | inhibited VC | 165 |
| miR‐211 | mice | mmu-miR-211-5p | mmu-miR-211 | \ | \ | \ | >mmu-miR-211-5p MIMAT0000668 UUCCCUUUGUCAUCCUUUGCCU | \ | \ |
| miR-770-3p | mice | mmu-miR-770-3p | mmu-miR-770-3p | mouse VSMC line MOVAS-1 | β-glycerophosphate and pyruvic acid | up | >mmu-miR-770-3p MIMAT0003891 CGUGGGCCUGACGUGGAGCUGG | further researches required | 174 |
| miR-770-3p | rat | rno-miR-770-3p | rno-miR-770* | descending thoracic aorta | cystic kidney disease rats | up | >rno-miR-770-3p MIMAT0017317 GUGGGCCUGACGUGGAG | further researches required | 175 |
| miR-296-3p | mice | mmu-miR-296-3p | mmu-miR-296-3p | mouse VSMC line MOVAS-1 | β-glycerophosphate and pyruvic acid | up | >mmu-miR-296-3p MIMAT0004576 GAGGGUUGGGUGGAGGCUCUCC | further researches required | 174 |
| miR-296-3p | rat | rno-miR-296-3p | rno-miR-296 | descending thoracic aorta | cystic kidney disease rats | up | >rno-miR-296-3p MIMAT0004742 GAGGGUUGGGUGGAGGCUCUCC | further researches required | 175 |
| miR-702-5p | mice | mmu-miR-702-5p | mmu-miR-702-5p | mouse VSMC line MOVAS-1 | β-glycerophosphate and pyruvic acid | up | >mmu-miR-702-5p MIMAT0022931 GUGAGUGGGGUGGUUGGCAUG | further researches required | 174 |
| miR-702-5p | rat | rno-miR-702-5p | rno-miR-702-5p | descending thoracic aorta | cystic kidney disease rats | up | >rno-miR-702-5p MIMAT0017884 GUGAGUGGGGUGGUUGGCAUG | further researches required | 175 |
| miR-204-3p | mice | mmu-miR-204-3p | mmu-miR-204* | mouse VSMC line MOVAS-1 | β-glycerophosphate and pyruvic acid | up | >mmu-miR-204-3p MIMAT0017002 GCUGGGAAGGCAAAGGGACGU | further researches required | 174 |
| miR-204-3p | rat | rno-miR-204-3p | rno-miR-204* | descending thoracic aorta | cystic kidney disease rats | up | >rno-miR-204-3p MIMAT0004739 GCUGGGAAGGCAAAGGGACGUU | further researches required | 175 |
| miR-667-5p | mice | mmu-miR-667-5p | mmu-miR-667* | mouse VSMC line MOVAS-1 | β-glycerophosphate and pyruvic acid | up | >mmu-miR-667-5p MIMAT0017239 CGGUGCUGGUGGAGCAGUGAGCACG | further researches required | 174 |
| miR-667-5p | rat | rno-miR-667-5p | rno-miR-667* | descending thoracic aorta | cystic kidney disease rats | up | >rno-miR-667-5p MIMAT0017369 CGGUGCUGGUGGAGCAGUGAGCAC | further researches required | 175 |
| miR-92b-5p | mice | mmu-miR-92b-5p | mmu-miR-92b* | mouse VSMC line MOVAS-1 | β-glycerophosphate and pyruvic acid | up | >mmu-miR-92b-5p MIMAT0017278 AGGGACGGGACGUGGUGCAGUGUU | further researches required | 174 |
| miR-92b-5p | rat | rno-miR-92b-5p | rno-miR-92b* | descending thoracic aorta | cystic kidney disease rats | up | >rno-miR-92b-5p MIMAT0017319 AGGGACGGGACGCGGUGCAGUGUU | further researches required | 175 |
| miR-494-4p | mice | mmu-miR-494-3p | mmu-miR-494 | mouse VSMC line MOVAS-1 | β-glycerophosphate and pyruvic acid | up | >mmu-miR-494-3p MIMAT0003182 UGAAACAUACACGGGAAACCUC | further researches required | 174 |
| miR-494-3p | rat | rno-miR-494-3p | rno-miR-494 | descending thoracic aorta | cystic kidney disease rats | up | >rno-miR-494-3p MIMAT0003193 UGAAACAUACACGGGAAACCUCU | further researches required | 175 |
| miR-199a-5p | mice | mmu-miR-199a-5p | mmu-miR-199a | mouse VSMC line MOVAS-1 | β-glycerophosphate and pyruvic acid | down | >mmu-miR-199a-5p MIMAT0000229 CCCAGUGUUCAGACUACCUGUUC | further researches required | 174 |
| miR-199a-5p | rat | rno-miR-199a-5p | rno-miR-199a | descending thoracic aorta | cystic kidney disease rats | down | >rno-miR-199a-5p MIMAT0000872 CCCAGUGUUCAGACUACCUGUUC | further researches required | 175 |
| miR-19b-3p | mice | mmu-miR-19b-3p | mmu-miR-19b | mouse VSMC line MOVAS-1 | β-glycerophosphate and pyruvic acid | down | >mmu-miR-19b-3p MIMAT0000513 UGUGCAAAUCCAUGCAAAACUGA | further researches required | 174 |
| miR-19b-3p | rat | rno-miR-19b-3p | rno-miR-19b | descending thoracic aorta | cystic kidney disease rats | down | >rno-miR-19b-3p MIMAT0000788 UGUGCAAAUCCAUGCAAAACUGA | further researches required | 175 |
| miR-99b | mice | mmu-miR-99b-5p | mmu-miR-99b | mouse VSMC line MOVAS-1 | β-glycerophosphate and pyruvic acid | down | >mmu-miR-99b-5p MIMAT0000132 CACCCGUAGAACCGACCUUGCG | further researches required | 174 |
| miR-99b | rat | rno-miR-99b-5p | rno-miR-99b | descending thoracic aorta | cystic kidney disease rats | down | >rno-miR-99b-5p MIMAT0000821 CACCCGUAGAACCGACCUUGCG | further researches required | 175 |
| miR-24-3p | mice | mmu-miR-24-3p | mmu-miR-24 | mouse VSMC line MOVAS-1 | β-glycerophosphate and pyruvic acid | down | >mmu-miR-24-3p MIMAT0000219 UGGCUCAGUUCAGCAGGAACAG | further researches required | 174 |
| miR-24-3p | rat | rno-miR-24-3p | rno-miR-24 | descending thoracic aorta | cystic kidney disease rats | down | >rno-miR-24-3p MIMAT0000794 UGGCUCAGUUCAGCAGGAACAG | further researches required | 175 |
| let-7b-5p | mice | mmu-let-7b-5p | mmu-let-7b | mouse VSMC line MOVAS-1 | β-glycerophosphate and pyruvic acid | down | >mmu-let-7b-5p MIMAT0000522 UGAGGUAGUAGGUUGUGUGGUU | further researches required | 174 |
| let-7b-5p | rat | rno-let-7b-5p | rno-let-7b | descending thoracic aorta | cystic kidney disease rats | down | >rno-let-7b-5p MIMAT0000775 UGAGGUAGUAGGUUGUGUGGUU | further researches required | 175 |
| miR-30e-5p | mice | mmu-miR-30e-5p | mmu-miR-30e | mouse VSMC line MOVAS-1 | β-glycerophosphate and pyruvic acid | down | >mmu-miR-30e-5p MIMAT0000248 UGUAAACAUCCUUGACUGGAAG | further researches required | 174 |
| miR-30e-5p | rat | rno-miR-30e-5p | rno-miR-30e | descending thoracic aorta | cystic kidney disease rats | down | >rno-miR-30e-5p MIMAT0000805 UGUAAACAUCCUUGACUGGAAG | further researches required | 175 |
